# Towards a generic physiologically based kinetic model to predict in vivo uterotrophic responses in rats by reverse dosimetry of in vitro estrogenicity data

**DOI:** 10.1007/s00204-017-2140-5

**Published:** 2017-12-12

**Authors:** Mengying Zhang, Bennard van Ravenzwaay, Eric Fabian, Ivonne M. C. M. Rietjens, Jochem Louisse

**Affiliations:** 1 Division of Toxicology, Wageningen University, Stippeneng 4, 6708 WE Wageningen, The Netherlands; 2 Experimental Toxicology and Ecology, BASF SE, Z 470, 67056 Ludwigshafen, Germany

**Keywords:** Physiologically based kinetic modelling, Reverse dosimetry, In vitro–in vivo extrapolation, Uterotrophic assay, 17β-estradiol, Bisphenol A

## Abstract

Physiologically based kinetic (PBK) modelling-based reverse dosimetry is a promising tool for the prediction of in vivo developmental toxicity using in vitro concentration–response data. In the present study, the potential of this approach to predict the dose-dependent increase of uterus weight in rats upon exposure to estrogenic chemicals was assessed. In vitro concentration–response data of 17β-estradiol (E2) and bisphenol A (BPA) obtained in the MCF-7/BOS proliferation assay, the U2OS ER-CALUX assay and the yeast estrogen screen (YES) assay, were translated into in vivo dose–response data in rat, using a PBK model with a minimum number of in vitro and in silico determined parameter values. To evaluate the predictions made, benchmark dose (BMD) analysis was performed on the predicted dose–response data and the obtained BMDL_10_ values were compared with BMDL_10_ values derived from data on the effects of E2 and BPA in the uterotrophic assay reported in the literature. The results show that predicted dose–response data of E2 and BPA matched with the data from in vivo studies when predictions were made based on YES assay data. The YES assay-based predictions of the BMDL_10_ values differed 3.9-fold (E2) and 4.7- to 13.4-fold (BPA) from the BMDL_10_ values obtained from the in vivo data. The present study provides the proof-of-principle that PBK modelling-based reverse dosimetry of YES assay data using a minimum PBK model can predict dose-dependent in vivo uterus growth caused by estrogenic chemicals. In future studies, the approach should be extended to include other estrogens.

**Electronic supplementary material:**

The online version of this article (10.1007/s00204-017-2140-5) contains supplementary material, which is available to authorized users.

## Introduction

The development, validation and application of reliable non-animal based approaches for the hazard and risk characterization of chemicals is urgently needed for modern twenty-first century toxicological risk assessment (Wetmore et al. 2011). To this end, many efforts in the field have focused on developing in vitro assays for toxicity studies enabling the process of risk assessment. However, concentration–response data obtained from such in vitro assays cannot directly be used for risk assessment, which requires dose–response data on adverse effects to obtain points of departure to set safe exposure levels in humans. Therefore, concentration–response data from in vitro assays need to be translated to predicted in vivo dose–response data to facilitate the use of in vitro toxicity data in toxicological risk assessment. This translation is feasible using so-called physiologically based kinetic (PBK) modelling-based reverse dosimetry (Coecke et al. 2013; Louisse et al. 2017; Wetmore et al. 2011).

We have shown this PBK modelling-based reverse dosimetry approach to adequately predict in vivo developmental toxicity for diverse chemicals using concentration–response data obtained in the embryonic stem cell test (Li et al. 2017; Louisse et al. 2010, 2015; Strikwold et al. 2013, 2017). To further develop the approach, proofs of principle are required for the prediction of in vivo dose–response data for other toxicological endpoints as well. Recently, we showed that in vivo dose-dependent nephrotoxicity of aristolochic acid could be adequately predicted using PBK modelling-based reverse dosimetry of in vitro toxicity data obtained in kidney cells, indicating that application of the approach for diverse toxicological endpoints seems feasible (Abdullah et al. 2016). In previous studies, the approach was developed for individual chemicals. To efficiently predict dose-dependent toxicity for (large) groups of chemicals, the development of a generic PBK modelling-based reverse dosimetry approach for large numbers of chemicals is required.

The present study aims to develop a combined in vitro-PBK modelling approach to predict the dose-dependent in vivo uterotrophic growth induced by (estrogenic) chemicals in rats using a minimum PBK model as a first step towards a generic PBK model for estrogenic chemicals. The uterotrophic assay is used as the primary in vivo test for the detection of estrogenic chemicals (Ashby 2001; Kim et al. 2005; Odum et al. 1997; Yamasaki et al. 2002). This assay measures the uterus weight increase induced by estrogen receptor agonists (Ashby and Tinwell 1998; Kim et al. 2005; Reel et al. 1996). The endogenous estrogen 17β-estradiol (E2) and the xenoestrogen bisphenol A (BPA) were selected as the model compounds in the current study. E2 shows a high estrogenic potency in both in vitro and in vivo studies (Kim et al. 2001; Segner et al. 2003). In contrast, BPA shows far less estrogenic potency in vitro and in vivo, needing 3- to 4-order of magnitude higher concentrations or doses to induce the same estrogenic effects as caused by E2 (Kim et al. 2001; Kolle et al. 2010; Segner et al. 2003; Wang et al. 2014). Concentration–response data for E2 and BPA can be derived from a wide range of in vitro estrogenicity assays, including the MCF-7/BOS proliferation assay, the U2OS estrogen receptor-mediated chemical-activated luciferase gene expression (ER-CALUX) assay and the yeast estrogen screen (YES) assay. A previous study showed that the in vitro proliferation assay and reporter gene assays (ER-CALUX and YES assay) showed a reasonably good correlation with the in vivo uterotrophic assay (Wang et al. 2014). However, this analysis was performed without taking the kinetics into consideration and did not provide points of departure that can be used in risk assessment. In the present study, the in vitro and in vivo data were combined via the developed minimum PBK models for E2 and BPA to reveal whether PBK modelling-based reverse dosimetry of in vitro estrogenicity data can be used to predict in vivo uterotrophic growth, serving as a starting point to develop a generic PBK modelling-based reverse dosimetry approach that can be used for risk assessments of large groups of estrogenic chemicals.

## Materials and methods

### Materials

17β-estradiol (E2), bisphenol A (BPA), reduced nicotinamide adenine dinucleotide phosphate (NADPH), uridine 5′-diphosphoglucuronic acid (UDPGA), adenosine 3′-phosphate 5′-phosphosulfate (PAPS) lithium salt hydrate, acetyl coenzyme A (acetyl CoA) sodium salt, alamethicin, magnesium chloride, sodium phosphate, sodium chloride and rat serum were purchased from Sigma–Aldrich (Zwijndrecht, the Netherlands). Dimethyl sulfoxide (DMSO) was purchased from Acros Organics (Geel, Belgium) and phosphate-buffered saline (PBS) was purchased from Invitrogen (Breda, the Netherlands). Pooled liver S9 fractions from male and female Sprague–Dawley rats were obtained from Tebu-bio (Heerhugowaard, the Netherlands). Rapid equilibrium dialysis (RED) devices, including RED inserts, RED base plate and sealing tape, were purchased from Thermo Fisher Scientific (Bleiswijk, the Netherlands).

### Methods

#### PBK modelling-based reverse dosimetry approach

The PBK modelling-based reverse dosimetry approach (Louisse et al. 2017) was used to predict dose-dependent uterotrophic growth induced by the endogenous estrogen E2 and the xeno-estrogen BPA. The PBK modelling-based reverse dosimetry approach applied in the current study includes 5 steps: (1) development of PBK models that describe E2 and BPA kinetics in rats, (2) PBK model evaluation, (3) determination of in vitro effect concentrations of E2 and BPA in in vitro estrogenicity assays, (4) translation of in vitro concentration–response data into in vivo dose–response data using the PBK models developed, and (5) evaluation of the predicted dose-dependent estrogenic effects, including BMD analysis of predicted dose–response data and the in vivo dose–response data obtained from the literature.

##### Development of PBK models that describe E2 and BPA kinetics in rats

**Fig. 1:**
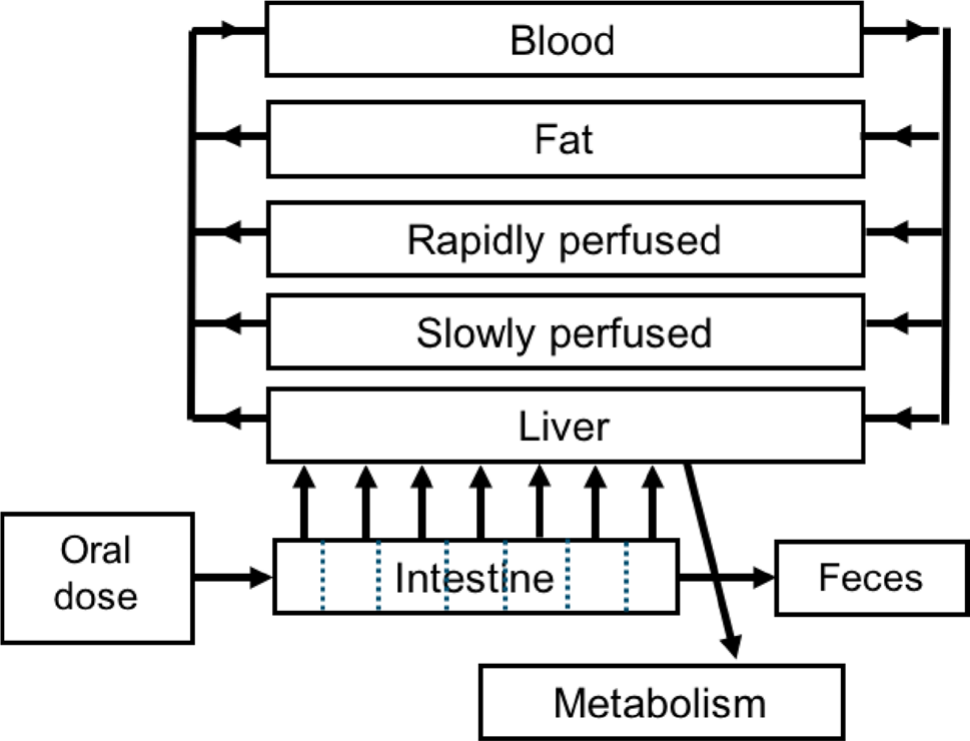
Schematic representation of the PBK model for E2 and BPA

To develop the PBK models that describe the kinetics of E2 and BPA in rats, we used our previously developed PBK model of tebuconazole (Li et al. 2017) as a starting point. A schematic representation of the model is shown in Fig. 1. The model included separate compartments for blood, liver, fat, rapidly perfused tissue and slowly perfused tissue. Additionally, a compartment for the intestines was included, which was divided into 7 sub-compartments, to describe the intestinal transition of the chemicals. The values for physiological and anatomical parameters were taken from literature (Brown et al. 1997), and are presented in supplementary material 1 Table 1.

The PBK model describes the in vivo kinetics upon intravenous injection or oral exposure. Intravenous exposure was included as exposure route to evaluate the PBK model prediction using the in vivo kinetic data available upon intravenous dosing. For oral exposure, stomach emptying and transition in the small intestine were included.

The apparent permeability coefficient (*P*_app_) values from Caco-2 transport studies were used to describe the uptake of E2 and BPA from the intestines to the liver for the 7 sub-compartments (Li et al. 2017; Louisse et al. 2015). The *P*_app_ value in the Caco-2 model (*P*_app, Caco−2_) is reported to be 17 × 10^−6^ cm/s for E2 (Yazdanian et al. 1998) and 20 × 10^−6^ cm/s for BPA (Yoshikawa et al. 2002). In vivo *P*_app_ values (*P*_app, in vivo_) were estimated based on the *P*_app, Caco−2_ value, using the formula Log (*P*_app, in vivo_) = 0.6836 × Log (*P*_app, Caco−2_) − 0.5579 (Sun et al. 2002), and applied in the PBK models as described by Li et al. (2017). Model parameter values for the intestinal absorption of E2 and BPA were determined with the following equation: absorption rate (µmol/h) = *P*_app, in vivo_ (cm/h) × surface area of the rat small intestine (cm^2^) × luminal concentration of parent compound (mM) (Li et al. 2017; Louisse et al. 2015; Verwei et al. 2006). The luminal concentration of E2 and BPA in each sub-compartment was calculated by the model by dividing the amount of parent compound by the volume of each sub-compartment.

The partition coefficients of E2 and BPA in rats were estimated based on the quantitative property–property relationship (QPPR) approach of DeJongh et al. (1997). The chemical-dependent input parameter of this approach was the octanol–water partition coefficient (*P*_ow_). The Log *P*_ow_ values are 4.01 and 3.32 for E2 and BPA, respectively (Hansch et al. 1995). The calculated partition coefficients of E2 and BPA are presented in supplementary material 1 Table 2.

To determine parameter values for hepatic metabolism, in vitro incubations with rat liver S9 fractions were performed, as described below. We assumed that estrogenic effects of E2 and BPA are only caused by the parent compounds and not by the metabolites. Therefore, the PBK model only describes the kinetics of the parent compounds and not of the metabolites. Clearance of the parent compound was assumed to result from metabolic clearance only, and not from renal clearance. The model equations were coded and numerically integrated in Berkeley Madonna 8.0.1 (UC Berkeley, CA, USA), using the Rosenbrock’s algorithm for stiff systems.

*Determination of model parameter values for hepatic clearance*.The hepatic clearance (CL_int_) of E2 and BPA was determined to describe the clearance of the parent compounds in the PBK model. To develop a generic model, a fast and efficient approach needs to be applied to determine the hepatic clearance. To this end, a substrate depletion approach was used, as described by others (Jones and Houston 2004; Obach 1999; Obach and Reed-Hagen 2002). Metabolic breakdown of the parent compound, referred to as “substrate depletion” is used to measure in vitro CL_int_ of the parent compound, where the consumption rate of the parent compound using hepatic microsomes, hepatic S9 fractions or hepatocytes, is measured over a range of incubation times under linear conditions with respect to protein concentration and substrate concentration (Jones and Houston 2004). The advantage of this approach is that it does not require prior knowledge of metabolic pathways nor the quantification of metabolites (Obach 1999; Obach and Reed-Hagen 2002). A prerequisite of the approach is that the initial concentration of parent compound is below the Michaelis constant (*K*_m_). In the current study, the test concentrations of E2 and BPA were 3 μM, which is at least six to ninefold below the lowest *K*_m_ values reported for hydroxylation or glucuronidation of E2 and BPA (Alkharfy and Frye 2002; Brueggemeier 1981; Bui et al. 1990; Coughlin et al. 2012; Elsby et al. 2001; Verner et al. 2010).

The CL_int_ of the parent compounds via phase I and phase II metabolism was determined in incubations with liver S9 fraction from both male and female Sprague–Dawley rats, in the presence of all relevant co-factors NADPH, UDPGA, PAPS and acetyl CoA. To investigate whether the in vitro CL_int_ value derived from incubations for individual reactions (with individual co-factors) is similar as the CL_int_ value derived from incubations with all the co-factors together in one mixture, two approaches were applied. In the first approach, the parent compound was incubated with individual co-factors in Eppendorf tubes applying a final volume of 200 µL containing either 3 mM NADPH, 5 mM UDPGA (also including 5 mM MgCl_2_ and 0.025 mg/ml alamethicin), 0.2 mM PAPS, or 0.5 mM acetyl CoA, with 0.5 mg/ml liver S9 in 0.1 M potassium phosphate (pH 7.4) buffer. After pre-incubating the mixtures at 37 °C for 1 min, the reactions were started by adding 3 µM (final concentration) of the parent compound from a 100 times concentrated stock solution dissolved in DMSO. In the second approach, it was tested whether the methodology could be simplified by adding all the co-factors to one incubation mixture and the parent compound after pre-incubation. For both approaches, 14 time points were selected at which the reaction was terminated, being 0, 1, 2, 3, 4, 5, 7, 8.5, 10, 15, 20, 25, 30 and 45 min. To terminate the reaction 100 µL cold acetonitrile (ACN) was added to the mixture and the Eppendorf tubes were put on ice for 30 min. Subsequently, tubes were centrifuged at 15,000 rpm for 5 min (CT 15RE, Hitachi Koki Co., Ltd) and the supernatant was collected for UPLC analysis. For each incubation time point, a corresponding control was included, which was an incubation performed in the absence of co-factor(s). All incubations were performed in triplicate in three independent studies.

The concentration of the parent compound was quantified for all the incubations using UPLC analysis performed as described below. The ratio of remaining parent compound concentration between incubation sample (*C*_compound_) and the control without co-factor (*C*_control_) was calculated for each incubation time. Subsequently, the depletion curve of the parent compound [ln(*C*_compound_/*C*_control_)] against time was derived as described before (Jones and Houston 2004; Obach 1999; Obach and Reed-Hagen 2002). The slope of the linear part of the depletion curve represents the elimination rate constant (*k*, min^−1^) for elimination of the parent compound. Using the following equation, the in vitro clearance (CL_int, in vitro_) of the parent compound can be estimated: CL_int, in vitro_ (µL/min/mg protein) = V (µL)/*P* (mg) × *k* (min^−1^) (Obach 1999; Sjögren et al. 2009). V represents volume (µL) of incubation mixture and P is the protein amount (mg) in the mixture. The in vitro CL_int_ value derived from incubations with all co-factors together in one mixture (approach 2) was compared with the sum of the CL_int_ values derived from incubations with a single co-factor (approach 1). The in vitro CL_int_ of the parent compound was scaled to a whole liver, assuming that the S9 protein concentration in the rat liver is 87 g/kg liver (Chiu and Ginsberg 2011).

*Ultra-Performance Liquid Chromatography (UPLC) analysis*.The UPLC method for detection and quantification of E2 was adapted from Cai et al. (2009). For BPA, the analytical method was slightly changed based on the method for E2. In this study, a UPLC H_Class system (Waters Acquity) equipped with a Waters BEH C18 (1.7 µm, 2.1 × 50 mm) column was used to determine the AUC of the parent compounds. The temperature was set at 40 °C for the column and 5 °C for the samples. 3.5 µL supernatant of each incubation was injected into the system. Nano-pure water was used as mobile phase A and ACN was used as mobile phase B. For E2, the flow rate was set at 0.6 ml/min and the starting condition of the eluents was 90%:10% (V:V, A:B). The gradient changed into 36%:64% in 4 min, and subsequently returned to the initial condition in the next minute, and was kept for another minute before the next injection, with a total running time of 6 min. For BPA, the flow rate was set at 0.4 ml/min and the starting condition of the eluents (A:B) was 80%:20%. The gradient changed into 25%:75% in 2.4 min and changed into 0%:100% at 2.8 min. Subsequently, the gradient returned to the initial condition from 2.8 to 3.5 min which was kept until 4 min. The concentration of parent compound present in each incubation mixture was quantified using a calibration curve, made with commercially available reference compounds and using an absorption wavelength of 200 nm for E2 and 227 nm for BPA.

##### PBK model evaluation

To evaluate the performance of the PBK model developed, the time-dependent predicted blood concentrations of parent compounds were compared to the reported time-dependent blood concentrations of the parent compounds in rats upon exposure to different doses of E2 or BPA. To that end, a literature study on in vivo kinetic studies of E2 and BPA from rats upon intravenous (IV) and oral administration was performed using rats, single dose, intravenous injection or oral administration as the search terms.

A sensitivity analysis was performed to identify the most influential parameters of the model on the model prediction of the maximum blood concentration (*C*_max_) of the parent compound. Normalized sensitivity coefficients (SC) were calculated according to the following equation: SC = (*Cʹ* – *C*)/(*Pʹ* – *P*) × (*P*/*C*), where *C* represents the initial value of the model output and *Cʹ* is the modified value after changing the parameter value, *P* is the initial parameter value and *Pʹ* represents the modified parameter value (Evans and Andersen 2000; Waters et al. 2008). A 5% increase of parameter value was chosen to assess the effect of a change in parameter on the prediction of the *C*_max_. The sensitivity analysis was conducted for oral exposure to a single dose of 0.02 mg/kg bw E2 or 10 mg/kg bw BPA, which were the doses applied in the in vivo kinetic studies that were used for the model evaluation (Bawarshi-Nassar et al. 1989; Pottenger et al. 2000). Each parameter was analyzed individually by changing one parameter at a time and keeping the other parameters the same.

##### Determination of in vitro effect concentrations of E2 and BPA in different in vitro estrogenicity assays

Several in vitro assays are available that can be used to assess the in vitro estrogenic potency of chemicals. In the present study, data from three different in vitro assays were used for PBK modelling-based reverse dosimetry. These include a cell proliferation assay based on the human MCF-7/BOS breast cancer cell line and estrogen reporter (ER) gene assays based on human U2OS cells (ER-CALUX assay) and yeast cells (YES assay). Both reporter gene assays contain human ERs and measure an estrogenic response by the induction of the expression of a reporter gene that is coupled to an estrogen response element (ERE), resulting from the formation of ligand-ER complexes which activate ERE-mediated gene transcription (Kinnberg 2003; Sonneveld et al. 2006; Zacharewski 1997). In the YES assay, the yeast is also transformed with the expression plasmids carrying the reporter gene lac-Z which is encoding the enzyme β-galactosidase (Kolle et al. 2010). The proliferation assay detects estrogenic activity by measuring an increase in proliferation of MCF-7/BOS cells which is mediated via ERs (Desaulniers et al. 1998; Wang et al. 2012; Zacharewski 1997). Data of E2 and BPA in these in vitro assays were obtained from different studies from the literature (Kolle et al. 2010; Wang et al. 2012, 2014).

The in vitro responses were expressed as percentage of the maximum response of the chemicals. To this end, the maximum response that the chemical induced was set as 100%, and the response of each exposure concentration was calculated as a percentage of this maximum response. The obtained curves were fitted with a symmetrical sigmoidal model (Hill slope) in GraphPad Prism 5 (GraphPad Software Inc., San Diego, CA, USA).

##### Translation of in vitro concentration–response data into in vivo dose–response data using the PBK models developed

For each in vitro concentration–response dataset from the three assays, the PBK modelling-based reverse dosimetry approach was used to predict the dose levels that are required to reach concentrations in blood that were applied in these assays. As it is assumed that the fraction unbound (*f*_ub_) of the parent compound induces the toxicity, the possible difference in the *f*_ub_ in the in vitro assay medium and in rat serum was corrected prior to applying reverse dosimetry. The *f*_ub_ of E2 and BPA were determined for in vitro assay media and rat serum as described below.

*Determination of fraction unbound (f*_*ub*_ *) of E2 and BPA in rat serum and in in vitro medium*. In this study, the rapid equilibrium dialysis (RED) assay was performed to determine the *f*_ub_ of E2 and BPA (van Liempd et al. 2011; Waters et al. 2008; Wetmore et al. 2011). For the YES assay, the assay medium does not contain protein, so there is no need to determine the *f*_ub_ in this assay medium (*f*_ub_ = 1). The assay medium used in the ER-CALUX and the proliferation assay contains 5% FCS, so the RED assay was performed using the assay medium for these two assays and for rat serum.

The *f*_ub_ was determined using the protocol described by Waters et al. (2008). The RED device insert has two chambers, a plasma and a buffer chamber. In brief, a sample of 300 µL containing 5 µM of E2 or BPA in the in vitro medium or rat serum was added to the plasma chamber, and 500 µL of PBS was added to the buffer chamber. After incubating for 5 h at 37 °C at 250 rpm on an orbital shaker, the system reaches equilibrium (van Liempd et al. 2011). 25 µL of post-dialysis samples were collected from the plasma and buffer chambers in separate tubes. Simultaneously, 25 µL of corresponding buffer (PBS) was added to the sample taken from the plasma chamber and the same volume of corresponding rat serum or in vitro medium was added to the sample taken from the buffer chamber. Then, 300 µL cold precipitation buffer (90/10 ACN/water) was added to both samples to precipitate the proteins and release the compound. The samples were put on ice for 30 min and subsequently centrifuged for 15 min at 15,000×*g* (CT 15RE, Hitachi Koki Co., Ltd). Then, the supernatants were collected for UPLC analysis. By determining the compound concentration in each chamber, the fraction unbound can be calculated with the following equation: *f*_ub_ = concentration in buffer chamber/concentration in plasma chamber (van Liempd et al. 2011; Waters et al. 2008). The measurements were performed in triplicate in three independent studies.

*PBK modelling-based reverse dosimetry*. In vivo dose-dependent uterotrophic growth was assumed to depend on the maximum concentration (*C*_max_) of E2 and BPA reached in the blood. Consequently, for reverse dosimetry, the in vitro unbound concentration (*C*_ub, in vitro_) was set equal to the unbound *C*_max_ in blood (*C*_ub, in vivo_) to determine the dose that is required to cause an in vivo estrogenic effect. The *C*_ub, in vitro_ was estimated based on the equation: *C*_ub, in vitro_ = *C*_in vitro_ × *f*_ub, in vitro_, where *C*_in vitro_ is the nominal concentration applied in the in vitro assay and *f*_ub, in vitro_ is the fraction unbound of the chemical in the in vitro assay medium as determined using the RED assay. The *C*_ub, in vivo_ was estimated based on the equation: *C*_ub, in vivo_ = *C*_in vivo_ × *f*_ub, in vivo_, where *C*_in vivo_ is the nominal blood concentration in rat and f_ub, in vivo_ is the f_ub_ in rat serum. Assuming that for the prediction of in vivo toxicity, the *C*_ub, in vitro_ is the same as *C*_ub, in vivo_, the nominal blood concentration in rat can be described by the following equation: *C*_in vivo_ = *C*_ub, in vivo_/*f*_ub, in vivo_ = *C*_ub, in vitro_/*f*_ub, in vivo_ = (*C*_in vitro_ × *f*_ub, in vitro_)/f_ub, in vivo_. By calculating with the PBK model which dose is required to reach a *C*_in vivo_, an in vitro concentration can be translated into an in vivo dose. Performing this exercise for all the concentrations applied in the in vitro assays, the in vitro concentration–response curves were translated into predicted in vivo dose–response curves.

##### Evaluation of the predictions of dose-dependent estrogenic effects

To evaluate the potential of the combined in vitro-PBK modelling approach to predict in vivo dose–response data for in vivo estrogenic effects of E2 and BPA, the predicted dose–response data were compared with the dose–response data derived from in vivo uterotrophic assay studies. To that end, a literature research was performed using the search terms uterotrophic assay, rats, oral administration.

Furthermore, benchmark dose (BMD) values derived from the predicted dose–response data were compared with BMD values derived from the dose–response data from the uterotrophic assay as reported in the literature. To that end, BMD modelling was applied on dose–response data using the exponential model for continuous data of the PROAST software from The National Institute for Public Health and the Environment of the Netherlands (RIVM) version 38.9 (Slob 2002). The benchmark response (BMR) was defined as a 10% increase of response change compared to the control. The predicted dose level with an estimated 10% extra risk obtained is shown as BMD_10_. The lower and upper limits of the 95% confidence interval on the BMD_10_ are defined as BMDL_10_ and BMDU_10_. In PROAST, BMD values are only reported if the model fit of the data is accepted, so when the software provided BMD values, it was assumed that the data had been adequately modelled. We applied BMD modelling on the predicted dose–response data with the response presented as the percentage of the maximum response induced by E2 or BPA. For in vivo studies, the absolute uterus weight values were used as input for the response in the continuous model.

## Results

### Development of PBK models for E2 and BPA kinetics in rats

A schematic representation of the model is shown in Fig. 1. The model includes separate compartments for blood, liver, fat, rapidly perfused tissue and slowly perfused tissue. Additionally, a compartment for the intestines was included, which was divided in 7 sub-compartments, to describe intestinal transition of the chemicals. The values for physiological and anatomical parameters were taken from literature (Brown et al. 1997), and are presented in supplementary material 1 Table 1. The model code of the developed PBK model is presented in supplementary material 2.

#### Determination of model parameter values for hepatic clearance

In the PBK model, the clearance (CL_int_) of the parent compound was described by modelling its elimination in the liver. Table 1 presents the values of in vitro CL_int_ of E2 and BPA determined by incubating the parent compound with male rat liver S9 and individual co-factors. Table 2 displays the in vitro CL_int_ of E2 and BPA determined by incubating male or female rat liver S9 with all co-factors together in one incubation mixture. The depletion curves of the parent compounds of all experiments are presented in supplementary material 3.

**Table 1:** In vitro clearance (CL_int_) for E2 and BPA using male Sprague–Dawley rat liver S9 fraction incubated with individual co-factors

Compound	Co-factor	CL_int_ (µL/min/mg protein)
E2	NADPH	154.7 ± 26.7
	UDPGA	23.3 ± 0.9
	PAPS	4.4 ± 1.6
	Acetyl CoA	*
	SUM	182.4 ± 26.8
BPA	NADPH	60.0 ± 4.8
	UDPGA	339.7 ± 7.8
	PAPS	3.4 ± 2.1
	Acetyl CoA	*
	SUM	403.1 ± 9.4

* No CL_int_ value can be derived from the incubation

**Table 2:** In vitro clearance (CL_int_) for E2 and BPA using male or female Sprague–Dawley rat liver S9 fraction incubated with all co-factors (NADPH, UDPGA, PAPS, acetyl CoA) together in one mixture

Compound	Male CL_int_ (µl/min/mg protein)	Female CL_int_ (µl/min/mg protein)
E2	175 ± 13.3	65.5 ± 12.3
BPA	392 ± 6.7	431.6 ± 12.5

The substrate depletion approach was applied with male and female Sprague–Dawley rat liver S9, because the in vivo kinetic data used to evaluate the model prediction were obtained in both male and female rats. For E2, the sum of CL_int_ values derived from incubations of male liver S9 with individual co-factors was 182.4 ± 26.8 µl/min/mg protein, which is in line with the CL_int_ derived from incubations of male liver S9 with all co-factors together in one mixture, providing a value of 175 ± 13.3 µL/min/mg protein. For BPA the sum of the values of CL_int_ was 403.1 ± 9.4 µL/min/mg protein from incubations of male liver S9 with individual co-factors which is in line with the CL_int_ derived from incubations of male liver S9 with all co-factors together in one mixture, providing a value of 392 ± 6.7 µl/min/mg protein. This indicates that for both compounds, incubations with all co-factors together and with each co-factor separately provide similar clearance values. Therefore, in the following studies aiming to determine CL_int_ values for the female rat liver S9, incubations were only performed using all co-factors together. For E2, CL_int_ was 2.7-fold lower for female Sprague–Dawley rat liver S9 than for male Sprague–Dawley rat liver S9, whereas for BPA, CL_int_ for female Sprague–Dawley rat liver S9 was 1.1-fold higher than for male Sprague–Dawley rat liver S9.

### PBK model evaluation

**Fig. 2:**
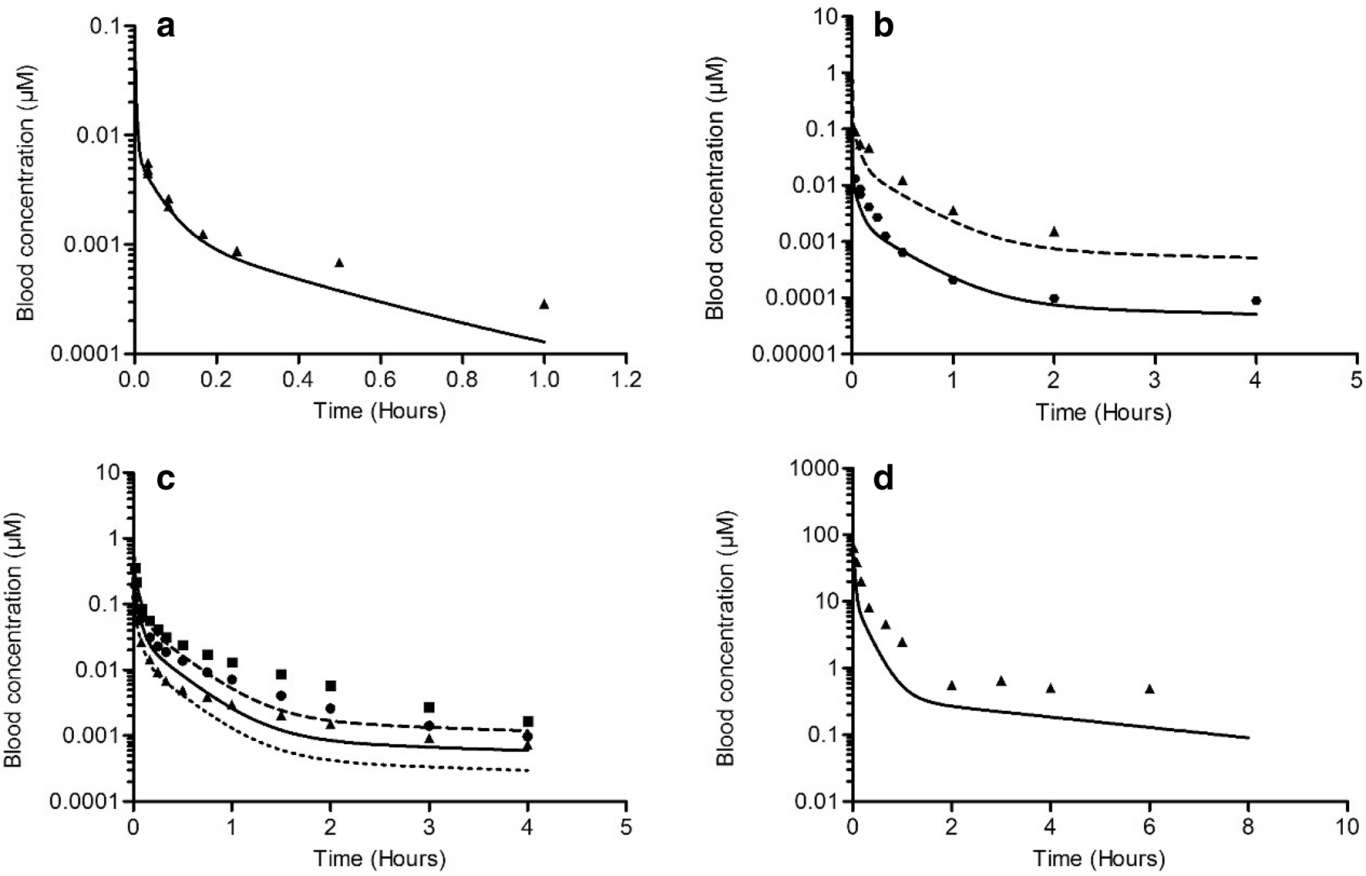
Blood concentrations of E2 (**a**–**c**) and BPA (**d**) in rats upon intravenous administration. Symbols represent the average blood concentrations from in vivo studies reported in the literature. Lines represent blood concentrations predicted by the PBK model. Dose levels are as follows:** a** E2, 0.0056 mg/kg bw (triangles, straight line) (Larner and Hochberg 1985);** b** E2, 0.01 mg/kg bw (circles, straight line) and 0.1 mg/kg bw (triangles, dashed line) (Eisenfeld 1967);** c** E2, 0.02 mg/kg bw (triangles, dotted line), 0.04 mg/kg bw (circles, straight line) and 0.08 mg/kg bw (squares, dashed line) (Bawarshi-Nassar et al. 1989);** d** BPA, 10 mg/kg bw (triangles, straight line) (Upmeier et al. 2000)

To evaluate the PBK model-based predictions, the predicted time-dependent blood concentrations of the parent compounds were compared with in vivo data on time-dependent blood concentrations reported in the literature (Fig. 2). The studies used for these evaluations are summarized in Table 3.

**Table 3:** Overview of studies from literature used to evaluate PBK model predictions

Compound	Species (strain)	Sex	Dose (mg/kg bw)	Exposure route	References
E2	Rat (Sprague–Dawley rats)	Male	0.02, 0.04, 0.08	Intravenous, oral	Bawarshi-Nassar et al. (1989)
E2	Rat (Sprague–Dawley rats)	Female	0.0056	Intravenous	Larner and Hochberg (1985)
E2	Rat (Sprague–Dawley rats)	Female	0.01, 0.1	Intravenous	Eisenfeld (1967)
BPA	Rat (Fisher 344)	Male	10, 100	Oral	Pottenger et al. (2000)
BPA	Rat (DA/Han rats)	Female	10	Intravenous	Upmeier et al. (2000)

**Fig. 3:**
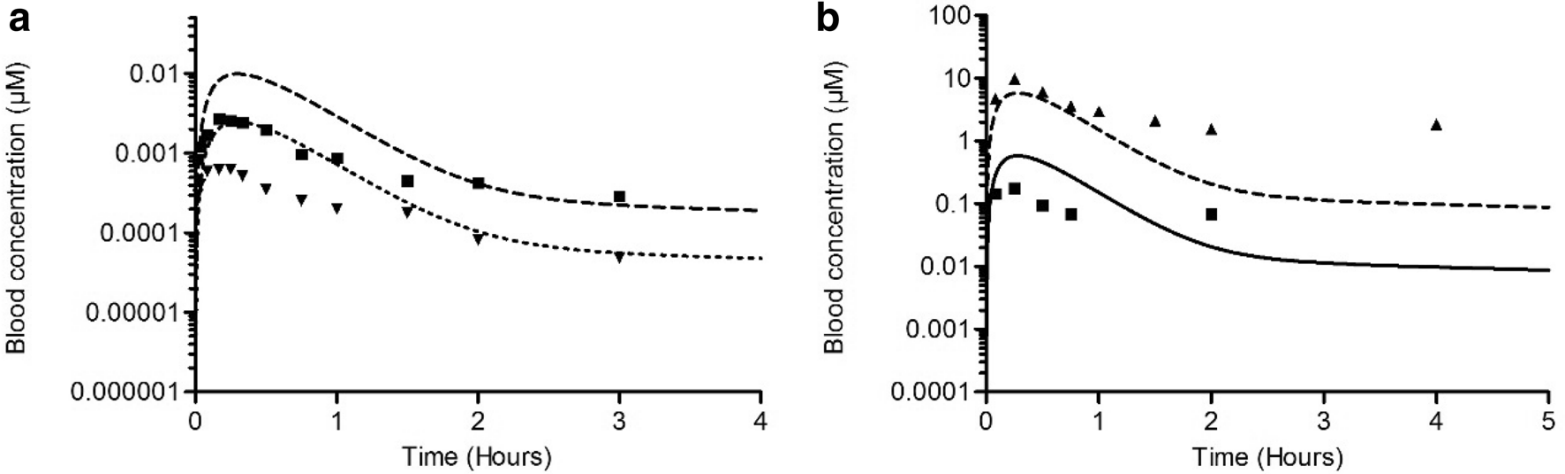
Blood concentrations of E2 (**a**) and BPA (**b**) in rats upon oral administration. Symbols represent the average blood concentrations from in vivo studies reported in the literature. Lines represent blood concentrations predicted by the PBK model. Dose levels are as follows:** a** E2, 0.02 mg/kg bw (triangles, dotted line) and 0.08 mg/kg bw (squares, dashed line) (Bawarshi-Nassar et al. 1989);** b** BPA, 10 mg/kg bw (squares, straight line) and 100 mg/kg bw (triangles, dashed line) (Pottenger et al. 2000)

Figure 2 displays the blood concentrations of E2 and BPA reported in the in vivo kinetic studies (symbols), and the PBK model-predicted blood concentrations (lines) upon IV administration. The comparison indicates that the developed PBK model accurately predicts the E2 or BPA blood concentrations upon IV exposure at various dose levels. Figure 3 shows the blood concentrations of E2 and BPA reported in the in vivo kinetic studies (symbols), and the PBK model-predicted blood concentrations (lines) upon oral dosing. The prediction of in vivo blood concentrations upon oral dosing was considered sufficiently adequate for a PBK model solely developed based on in vitro- and in silico-derived input parameter values, and the difference of *C*_max_ between the in vivo data and model prediction was at 1.7- to 4.2-fold (Table 4).

**Table 4:** *C*_max_ of E2 and BPA obtained from in vivo data and predicted by the PBK model in rats upon oral administration

Compound	Dose (mg/kg bw)	In vivo *C*_max_ (µM)	Predicted *C*_max_ (µM)	Predicted *C*_max_/in vivo *C*_max_	References
E2	0.02	0.0006	0.0025	4.17	Bawarshi-Nassar et al. (1989)
E2	0.08	0.0027	0.0098	3.63	Bawarshi-Nassar et al. (1989)
BPA	10	0.1748	0.5833	3.34	Pottenger et al. (2000)
BPA	100	9.8774	5.8334	0.59	Pottenger et al. (2000)

#### Sensitivity analysis

**Fig. 4:**
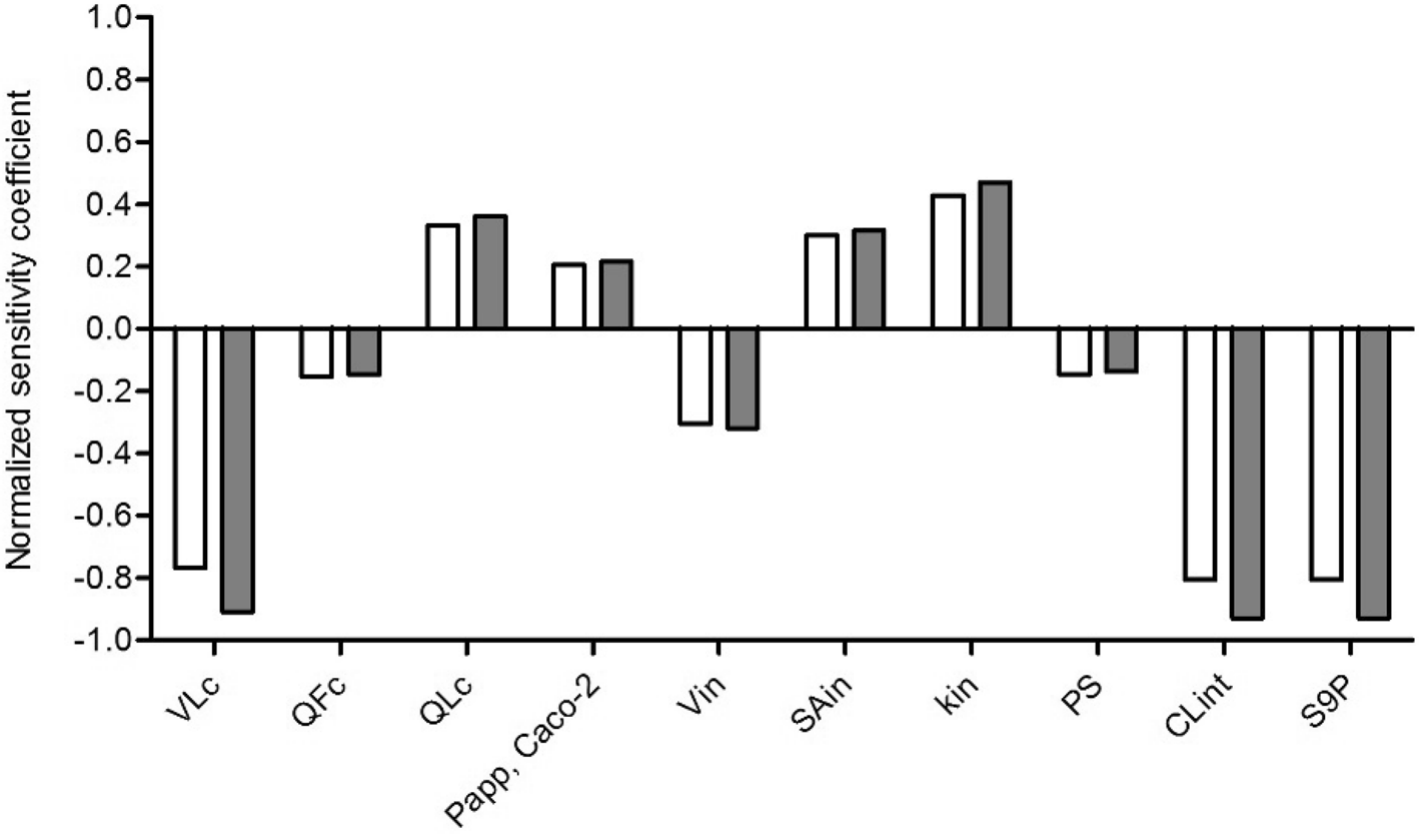
Normalized sensitivity coefficients of PBK model parameters for the predicted *C*_max_ of parent compound in blood after oral administration of 0.02 mg/kg bw E2 (white bars) or 10 mg/kg bw BPA (dark grey bars). All model parameters with normalized sensitivity coefficients with an absolute value higher than 0.1 are shown. VLc = fraction of liver tissue, QFc = fraction of blood flow to fat, QLc = fraction of blood flow to liver, *P*_app_, Caco-2 = *P*_app_ valued derived from Caco-2 transport studies, Vin = intestine volume for intestinal sub-compartment, SAin = intestinal surface area for intestinal sub-compartment, kin = transfer rate within intestinal sub-compartments, PS = slowly perfused tissue/blood partition coefficient, CL_int_ = experimental hepatic clearance of parent compound, S9P = S9 protein concentration in rat liver

Sensitivity analyses for the prediction of the *C*_max_ in blood upon exposure to a single oral dose of 0.02 mg/kg bw E2 or 10 mg/kg bw BPA were performed. The parameters with the absolute value of normalized sensitivity coefficient (SC) higher than 0.1 are presented in Fig. 4. The results indicate that the prediction of *C*_max_ of E2 or BPA in the PBK model is most sensitive to the parameters related to the liver, such as the hepatic clearance (CL_int_), protein scaling factor (S9 protein concentration in the rat liver, S9P) and fraction of liver tissue (VLc). The most influential chemical-dependent parameters are the *P*_app_ value and the hepatic clearance (CL_int_). All the sensitivity coefficients for the remaining model parameters are between − 0.1 and 0.1.

### Determination of in vitro effect concentrations of E2 and BPA in different in vitro estrogenicity assays

**Fig. 5:**
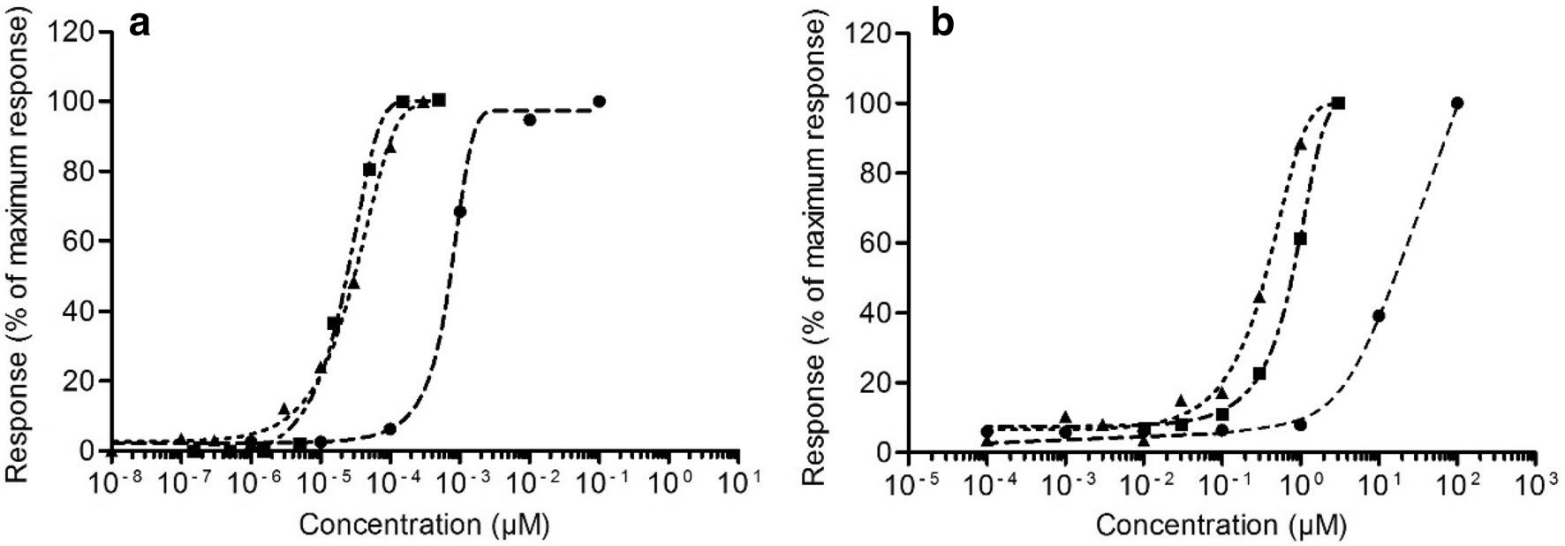
In vitro concentration-response curves of E2 (**a**) and BPA (**b**). The response is shown as a percentage of the maximum response induced by the chemical. Data derived from different in vitro assays shown with different symbols are as follows: MCF-7/BOS proliferation assay (triangles, dotted line), U2OS ER-CALUX assay (squares, dash-dotted line) and YES assay (circles, dashed line)

The in vitro data from the MCF-7/BOS proliferation assay, the ER-CALUX assay and the YES assay were taken from previous studies (Kolle et al. 2010; Wang et al. 2014). Figure 5 displays the data by presenting each response as a percentage of the maximum response of the chemical.

Different assays for estrogenicity show considerable differences in terms of potency. The MCF-7/BOS proliferation assay and the U2OS ER-CALUX assay show similar concentration–response curves for E2 and these assays also show similar concentration–response curves for BPA. In contrast, the YES assay is less sensitive than these assays, indicated by the 2–3 orders of magnitude higher concentrations that are required for E2 and BPA, respectively, to obtain an estrogenic response in the YES assay. E2 is 3–4 orders of magnitude more potent than BPA in all three assays, which is in line with previous studies (Fang et al. 2000; Gutendorf and Westendorf 2001).

### Translation of in vitro concentration–response data into in vivo dose–response data

#### Determination of unbound fraction (*f*_ub_) of parent compound in rat serum and in in vitro medium

Both E2 and BPA are highly bound to constituents in rat serum, indicated by a *f*_ub_ of 0.050 ± 0.005 and 0.040 ± 0.001, respectively. The *f*_ub_ of E2 and BPA in assay medium was 0.628 ± 0.012 and 0.461 ± 0.035, respectively. These data were used to correct for differences in free fraction of E2 and BPA in vitro compared to in vivo, as described in the “Materials and Methods” section.

#### PBK modelling-based reverse dosimetry

**Fig. 6:**
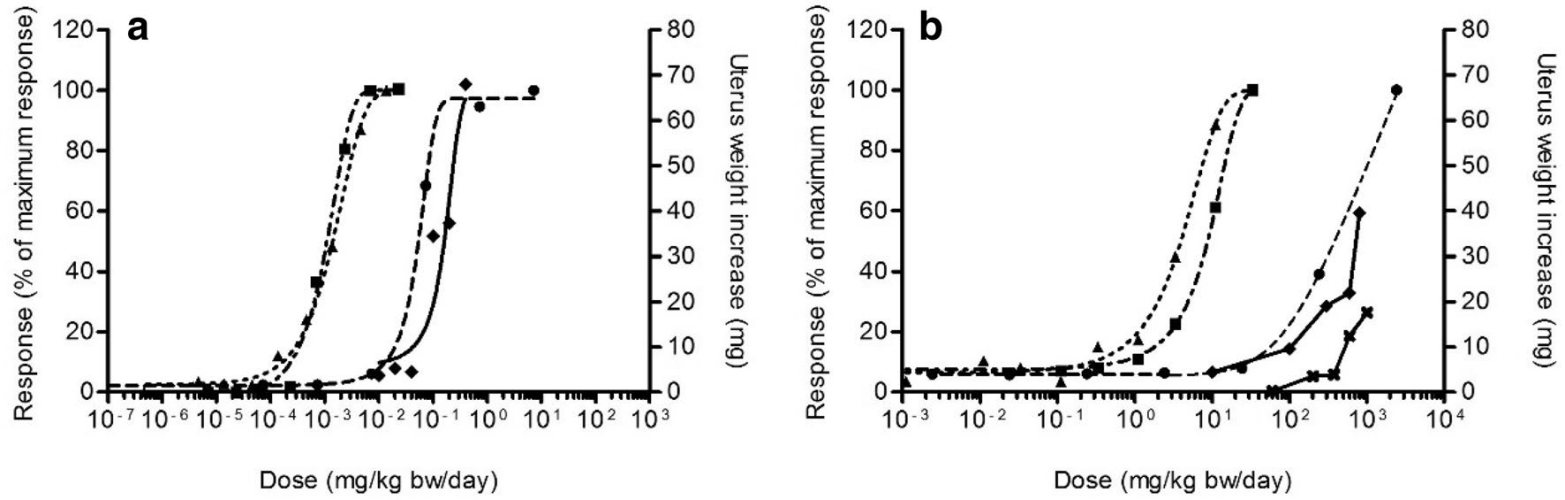
Predicted in vivo dose–response data in rats compared with in vivo data on uterotrophic growth upon exposure to E2 (**a**) and BPA (**b**). The predicted responses are shown as a percentage of maximum response induced by the chemical. Symbols represent the response derived from different in vitro assays, MCF-7/BOS proliferation assay (triangles, dotted line), U2OS ER-CALUX assay (squares, dash-dotted line), YES assay (circles, dashed line), or the absolute uterus weight increase of rats in the in vivo uterotrophic assay. For E2 (**a**), the in vivo data are from Odum et al. (1997) (diamonds, straight line). For BPA (**b**, **d**), in vivo data are from Tinwell and Ashby (2004) (diamonds, straight line) and from Kanno et al. (2003) (cross, straight line)

Using the PBK model and correction for differences in free fraction, the in vitro concentration–response curves for estrogenicity (shown in Fig. 5) were converted to in vivo dose–response curves. The predicted in vivo dose–response data obtained by this PBK modelling-based reverse dosimetry are presented as dotted line (based on MCF-7/BOS proliferation data), dash-dotted line (based on U2OS ER-CALUX data) and dashed line (based on YES data) in Fig. 6.

### Evaluation of the predictions of dose-dependent estrogenic effects

Table 5 presents the information of the in vivo uterotrophic assay studies of E2 and BPA that were used to evaluate the predicted in vivo dose–response data. The obtained dose–response data from the PBK modelling-based reverse dosimetry of the in vitro data were evaluated by comparing the predicted data with the in vivo data (Fig. 6). As already indicated above, for both E2 and BPA, the predictions based on the YES assay are best in line with the reported in vivo data, whereas the predictions based on the MCF-7/BOS proliferation assay and the ER-CALUX assay result in effective dose levels that are 2–3 orders of magnitude lower than the in vivo data. Since this qualitative analysis indicated that the predicted dose–response data based on the YES assay were most close to the in vivo data, these predicted dose–response data were used for BMD analysis to quantitatively compare the predictions with the in vivo data.

**Table 5:** Overview of in vivo uterotrophic assay data from literature used to evaluate predicted in vivo dose-dependent estrogenic effects based on PBK modelling-based reverse dosimetry of in vitro estrogenicity data

Compound	Species (strain)	Dose (mg/kg bw)	Exposure route	References
E2	Rat (AP)	0, 0.01, 0.02, 0.04, 0.1, 0.2, 0.4	Oral gavage	Odum et al. (1997)
BPA	Rat (Sprague–Dawley)	0, 60, 200, 375, 600, 1000	Oral gavage	Kanno et al. (2003)
BPA	Rat (AP)	0, 10, 100, 300, 600, 800	Oral gavage	Tinwell and Ashby (2004)

#### BMD analysis of predicted dose–response data and in vivo data

The predicted in vivo dose–response data based on the YES assay were used to quantitatively compare the predictions with the in vivo data on uterotrophic growth upon exposure to E2 and BPA. We applied BMD modelling using the PROAST software on predicted dose–response data (data presented in Fig. 6) and the absolute uterus weight values obtained from in vivo studies. The BMR was defined as a 10% increase compared to the negative control, and the BMD analysis provided BMD_10_, BMDL_10_ and BMDU_10_ values.

**Fig. 7:**
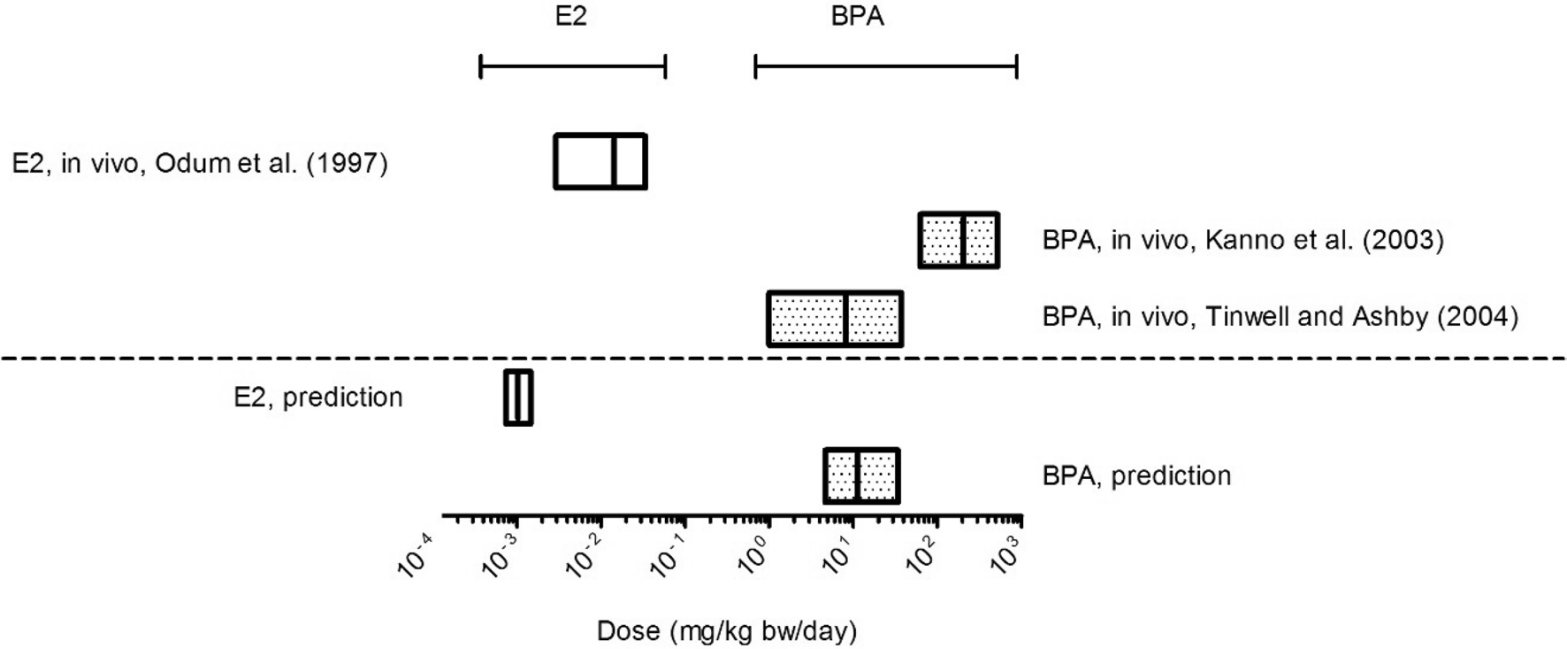
Comparison of the BMD_10_ values (including BMDL_10_ and BMDU_10_ values) from predicted dose–response and in vivo dose-response data of E2 (empty box) and BPA (dotted box). Predicted dose–response data were obtained by PBK modelling-based reverse dosimetry of in vitro data from the YES assay and in vivo data were obtained from uterotrophic assay studies reported in the literature (E2: (Odum et al. 1997), BPA: (Kanno et al. 2003) and (Tinwell and Ashby 2004)). BMD analysis was performed using the BMR as a 10% response change as compared to the control using PROAST. The results are presented as the range between the BMDL_10_ and BMDU_10_ values reported, giving the BMD_10_ values as straight line in between

The results of BMD analysis are displayed in Fig. 7. The range between BMDL_10_ and BMDU_10_ values is shown as a box, the BMD_10_ value is represented by the straight line in between. The predicted BMDL_10_ for E2 is 4 orders of magnitude lower than that of BPA while the in vivo data result in a BMDL_10_ for E2 that is 3–5 order of magnitude lower than that for BPA. For E2, the BMDL_10_ value derived from the predicted dose–response data was 3.9-fold lower than the BMDL_10_ value of the in vivo data. For BPA, the BMDL_10_ obtained from the predicted dose–response data was 4.7-fold higher than the BMDL_10_ value obtained from the vivo data from Tinwell and Ashby (2004) and 13.4-fold lower than the BMDL_10_ value obtained from the data of Kanno et al. (2003). Detailed information on the BMD analysis can be found in supplementary material 4.

## Discussion

The aim of present study was to assess whether in vivo dose-dependent estrogenic activity of chemicals, as measured by uterus weight increase in the uterotrophic assay in rats, can be predicted by a combined in vitro-PBK modelling approach using a simple and generic PBK model with a minimum number of parameters that can be defined in vitro and in silico. To that end, concentration–response data from three in vitro assays (MCF-7/BOS proliferation assay, U2OS ER-CALUX reporter gene assay and YES reporter gene assay) for estrogenic activities of E2 and BPA were translated to predicted in vivo dose–response data using a simple generic PBK model. The predicted data were compared with in vivo uterotrophic assay data of these compounds. The results reveal that predictions based on the YES reporter gene assay matched in vivo data on E2- and BPA-induced uterus weight increase best. The BMDL_10_ values obtained from these predicted dose–response data differed between 3.9-fold for E2 and 4.9- to 13.4-fold for BPA from BMDL_10_ values derived from data obtained from in vivo uterotrophic assay studies. From this study, it can be concluded that the use of the PBK modelling-based reverse dosimetry of in vitro data from the YES assay is promising to predict the in vivo dose-dependent uterus response of estrogenic chemicals.

In this study, we developed a simple PBK model based on in vitro and in silico derived parameter values describing the kinetics of the parent compounds. *P*_app_ values from Caco-2 transport studies were used to describe intestinal absorption and the logP values of the chemicals were used as input to calculate tissue:blood partition coefficients. Hepatic clearance was described as the only clearance process in the model. Although renal clearance may also play an important role in total clearance of chemicals from the body, this process was not included in the PBK model that was developed in the present study. This was done, since in vivo kinetic studies indicate that mainly metabolites are excreted in the urine (Busso and Ruiz 2011), suggesting that renal clearance does not play a major role in the clearance of the parent compounds. A substrate depletion approach based on incubations with hepatic S9 fractions was used for the determination of parameter values to describe the hepatic clearance of the parent compounds (Obach 1999; Obach and Reed-Hagen 2002). Although primary hepatocytes have been proven to provide an accurate and precise prediction of in vivo hepatic clearance (Brian Houston and Carlile 1997; Ito and Houston 2004), we have chosen to work with liver fractions, since hepatocytes are sensitive for disturbances and may, therefore, provide unreliable data in terms of reproducibility. For example, hepatocyte isolation often results in cell damage and hepatocytes show a loss of cytochrome P450 enzyme activity within 4–8 h after isolation (Hewitt et al. 2007; Soldatow et al. 2013). Since liver fractions are more easy to use compared to hepatocytes and liver slices, we used rat liver S9 fractions, which contain metabolic enzymes for both phase I and phase II reactions (Brandon et al. 2003), to determine the hepatic clearance of the parent compounds in the present study. The results showed that total hepatic clearance can be adequately determined using this approach applying co-factors of both phase I and phase II reactions at the same time, which was indicated by the fact that the obtained CL_int_ from an incubation of E2 or BPA containing rat liver S9 enzymes with the co-factors NADPH, UDPGA, PAPS and acetyl CoA provided a similar value as the sum of the CL_int_ values of incubations with individual co-factors. This indicates that for future studies with other chemicals, incubations with all co-factors in one mixture can be used to determine the value for hepatic clearance, which enables the efficient and reliable determination of parameter values for hepatic clearance.

The time-dependent blood concentrations predicted by the PBK model developed in the present study were in general in line with the blood concentrations of rats upon intravenous dosing as reported in in vivo studies. For two studies, the predicted blood concentrations were slightly lower than the blood concentrations observed in vivo (Fig. 2c, d). These differences were less than threefold. Given that the model parameter values were only derived from in vitro and in silico tools, and that no in vivo data were used to fit parameter values, we considered the PBK model predictions adequate and suitable for in vitro to in vivo extrapolations.

To develop a generic PBK model to predict the estrogenicity of a large number of chemicals using reverse dosimetry, the parameterization of the model should be fast and efficient. The sensitivity analysis revealed that the most influential chemical-specific parameters that determine the internal concentration in blood are the hepatic clearance and the *P*_app_ value, indicating the importance of the correct estimation of these parameter values. *P*_app_ values from experimental Caco-2 transport studies reported in the literature were used to describe the intestinal absorption, and the hepatic clearance of parent compounds were experimentally determined in the current study. However, to extend this PBK model to a generic model for a large number of chemicals, derivation of parameter values based on in silico methods is desirable. It has been shown that *P*_app_ values for Caco-2 transport can be predicted using QSAR approaches requiring information on the polar surface area alone (Hou et al. 2004) or combined with the hydrogen bonding capacity (van De Waterbeemd et al. 1996), indicating that no experimental studies are required to determine these parameter values. So far, no reliable QSAR approaches are available to estimate hepatic clearance, so this should be determined experimentally, for example using a substrate depletion approach as applied in the present study.

We translated in vitro concentration–response data from the U2OS ER-CALUX reporter gene assay, the MCF-7/BOS proliferation assay and the YES reporter gene assay to in vivo dose levels. Interestingly, the YES reporter gene assay requires 2–3 orders of magnitude higher concentrations of E2 and BPA, respectively, to reach estrogenic responses compared to the ER-CALUX reporter gene assay and the MCF-7/BOS proliferation assay. Figure 6 indicates that the predictions based on YES assay data match the in vivo dose-dependent uterus weight responses better than the predictions based on the other two assays. The possible explanation of lower sensitivity of the YES reporter gene assay compared to the sensitivity of the other assays might be related to the lower permeability through the thick cell walls of yeast cells compared to the mammalian cells resulting in lower intracellular concentrations (Zourob 2010). As predictions based on YES assay data were closer to in vivo data than predictions based on data from the other two assays, the relatively low intracellular concentrations in the YES assay may better reflect the in vivo situation than the relatively high intracellular concentrations in the other assays.

A BMD analysis was performed to quantify the effect levels of the predicted dose–response data and the in vivo dose–response data. The BMDL_10_ values obtained from the in vivo uterus weight studies differed 3.9-fold and 4.7- to 13.4-fold for E2 and BPA, respectively, from the BMDL_10_ values derived from the predicted dose–response data based on the YES assay, indicating that the PBK modelling-based reverse dosimetry of YES data is a promising approach to predict the dose-dependent uterus growth induced by estrogenic chemicals. It is also of interest to note the difference between the two in vivo studies for BPA revealing a 25.8-fold difference in BMD_10_ values. This suggests that the in vitro-PBK model-based approach may not differ from the in vivo data more than the variation also observed between different in vivo studies.

In conclusion, we showed that PBK modelling-based reverse dosimetry of in vitro concentration–response data from the YES reporter gene assay reasonably predicted in vivo dose-dependent uterus growth induced by E2 and BPA, showing the proof-of-concept of this approach for a novel toxicity endpoint. The current work serves as a starting point of developing a generic PBK model to predict the estrogenicity for a large number of chemicals. In our future work, we will assess whether dose-dependent uterus growth can be predicted for a larger set of estrogenic chemicals using this combined in vitro and PBK modelling-based reverse dosimetry approach. This will allow the evaluation whether the approach can reliably predict dose-dependent estrogenic effects of chemicals without the use of animal studies.

## Electronic supplementary material

Below is the link to the electronic supplementary material.

Supplementary material 1 (DOCX 17 KB)

Supplementary material 2 (DOCX 26 KB)

Supplementary material 3 (DOCX 375 KB)

Supplementary material 4 (DOCX 80 KB)
