# Supplementary material for: Towards a generic physiologically based kinetic model to predict in vivo uterotrophic responses in rats by reverse dosimetry of in vitro estrogenicity data
Source: Arch Toxicol. 2017 Dec 12;92(3):1075–88. doi: 10.1007/s00204-017-2140-5 (PMC5866837; doi:10.1007/s00204-017-2140-5)
Supplement: Supplementary file 1 — Supplementary material 1 (DOCX 17 KB) [file 204_2017_2140_MOESM1_ESM.docx]

Towards a generic physiologically based kinetic model to predict in vivo uterotrophic responses in rats by reverse dosimetry of in vitro estrogenicity data

Mengying Zhang^a*^, Bennard van Ravenzwaay^a,b^, Eric Fabian^b^, Ivonne M.C.M. Rietjens^a^, Jochem Louisse^a^

^a^ Division of Toxicology, Wageningen University, Stippeneng 4, 6708 WE Wageningen, the Netherlands

^b^ Experimental Toxicology and Ecology, BASF SE, Z 470, 67056 Ludwigshafen, Germany

^*^ Corresponding author: E-mail: [mengying.zhang@wur.nl](mailto:mengying.zhang@wur.nl); Tel: +31 317486396

**Supplementary material 1. Physiological parameter values and partition coefficients of E2 and BPA**

Table 1. Physiological parameter values used for the rat PBK model

| Physiological parameters | Values | Reference |
| --- | --- | --- |
| Body weight (kg) | 0.250 | Variable, dependent on study |
| *Tissue volumes*  *(fraction of body weight)* |  |  |
| Fat | 0.070 | Brown et al. (1997) |
| Liver | 0.034 | Brown et al. (1997) |
| Rapid perfused tissue | 0.056 | Brown et al. (1997) |
| Slowly perfused tissue | 67.6 | Brown et al. (1997) |
| Blood | 0.074 | Brown et al. (1997) |
| Cardiac output (L/h ∙ kg bw^0.74^) | 15 | Brown et al. (1997) |
| *Tissue blood flows*  *(fraction of cardiac output)* |  |  |
| Fat | 0.070 | Brown et al. (1997) |
| Liver | 0.25 | Brown et al. (1997) |
| Rapidly perfused tissue | 0.51 | Brown et al. (1997) |
| Slowly perfused tissue | 0.17 | Brown et al. (1997) |

Table 2. Partition coefficients of E2 and BPA used for the rat PBK model

| Parameters | Values |
| --- | --- |
| E2 |  |
| LogP_ow_ | 4.0^a^ |
| Fat/blood partition coefficient | 144^b^ |
| Liver/blood partition coefficient | 4.4 ^b^ |
| Rapid perfused tissue/blood partition coefficient | 4.4 ^b^ |
| Slowly perfused tissue/blood partition coefficient | 1.1 ^b^ |
|  |  |
| BPA |  |
| LogP_ow_ | 3.3^a^ |
| Fat/blood partition coefficient | 92 ^b^ |
| Liver/blood partition coefficient | 2.7 ^b^ |
| Rapid perfused tissue/blood partition coefficient | 2.7 ^b^ |
| Slowly perfused tissue/blood partition coefficient | 0.86 ^b^ |

^a^ Data from Hansch et al. (1995)

^b^ Partition coefficients were calculated using the QPPR method of DeJong et al. (1997).
