# Supplementary material for: Towards a generic physiologically based kinetic model to predict in vivo uterotrophic responses in rats by reverse dosimetry of in vitro estrogenicity data
Source: Arch Toxicol. 2017 Dec 12;92(3):1075–88. doi: 10.1007/s00204-017-2140-5 (PMC5866837; doi:10.1007/s00204-017-2140-5)
Supplement: Supplementary file 2 — Supplementary material 2 (DOCX 26 KB) [file 204_2017_2140_MOESM2_ESM.docx]

Towards a generic physiologically based kinetic model to predict in vivo uterotrophic responses in rats by reverse dosimetry of in vitro estrogenicity data

Mengying Zhang^a*^, Bennard van Ravenzwaay^a,b^, Eric Fabian^b^, Ivonne M.C.M. Rietjens^a^, Jochem Louisse^a^

^a^ Division of Toxicology, Wageningen University, Stippeneng 4, 6708 WE Wageningen, the Netherlands

^b^ Experimental Toxicology and Ecology, BASF SE, Z 470, 67056 Ludwigshafen, Germany

^*^ Corresponding author: E-mail: [mengying.zhang@wur.nl](mailto:mengying.zhang@wur.nl); Tel: +31 317486396

**Supplementary material 2. PBK model code of E2 and BPA**

2.1 PBK model for 17β-estradiol, built with in vitro and in silico data

Species: Rat

;=====================================================================

;Physiological parameters

;=====================================================================

;tissue volumes

BW = 0.250 {Kg} ; body weight rat (variable, dependent on study)

VFc = 0.07 ; fraction of fat tissue reference: (Brown et al., 1997).

VLc = 0.034 ; fraction of liver tissue reference: (Brown et al., 1997).

VBc = 0.074 ; fraction of blood reference: (Brown et al., 1997).

VRc = 0.09-VLc ; fraction of rapidly perfused tissue reference: (Brown et al., 1997).

VSc=0.746-VFc ; fraction of slowly perfused tissue reference: (Brown et al., 1997).

VF = VFc*BW {L or Kg} ; volume of fat tissue (calculated)

VL = VLc*BW {L or Kg} ; volume of liver tissue (calculated)

VB = VBc*BW {L or Kg} ; volume of blood (calculated)

VR = VRc*BW {L or Kg} ; volume of richly perfused tissue (calculated)

VS = VSc*BW {L or Kg} ; volume of slowly perfused tissue (calculated)

;--------------------------------------------------------------------------------------------------------------------

;blood flow rates

QC = 15*BW^0.74 {L/hr} ; cardiac output reference: (Brown et al., 1997).

QFc = 0.07 ; fraction of blood flow to fat reference: (Brown et al., 1997).

QLc = 0.25 ; fraction of blood flow to liver reference: (Brown et al., 1997).

QRc = 0.76 - QLc ; fraction of blood flow to rapidly perfused tissue reference: (Brown et al., 1997).

QSc = 0.24 – QFc ; fraction of blood flow to slowly perfused tissue reference: (Brown et al., 1997).

QF = QFc*QC {L/hr} ; blood flow to fat tissue (calculated)

QL = QLc*QC {L/hr} ; blood flow to liver tissue (calculated)

QR = QRc*QC {L/hr} ; blood flow to rapidly perfused tissue (calculated)

QS = QSc*QC {L/hr} ; blood flow to slowly perfused tissue (calculated)

;--------------------------------------------------------------------------------------------------------------------

; Intestinal lumen volumes, surfaces, absorption rates, transfer rates

;Papp, Caco-2 = 16.9 ; (x1E-6 cm/sec), in vitro, from Caco-2 reference: (Yazdanian et al. 1998)

PappCaco2=-4.77211 ; Log Papp, Caco-2

;Log (Papp,in vivo) = 0.6836*Log(PappCaco-2)-0.5579 reference: (Sun et al. 2002)

Papp=10^(0.6836*PappCaco2-0.5579)*3600/10

;apparent intestinal permeability coefficient in vivo {dm/hr}

Vin = 0.0012 ; volume for each compartment of intestines {L}

SAin = 0.134 ; surface area {dm2}

kin = 4.17 ; transfer rate to next compartment within the intestines {/hr}

;kabin1 = Papp*SAin ; absorption rate constant {L/hr}

Vin1 = Vin ; volume of intestine compartment 1 {L}

SAin1 = SAin ; surface area of intestine compartment 1 {dm2}

kabin1 = Papp*SAin1 ; absorption rate constant of intestine compartment 1 {L/hr}

kin1 = kin ; transfer rate to intestine compartment 2 {/hr}

Vin2 = Vin ; volume of intestine compartment 2 {L}

SAin2 = SAin ; surface area of intestine compartment 2 {dm2}

kabin2 = Papp*SAin2 ; absorption rate constant of intestine compartment 2 {L/hr}

kin2 = kin ; transfer rate to intestine compartment 3 {/hr}

Vin3 = Vin ; volume of intestine compartment 3 {L}

SAin3= SAin ; surface area of intestine compartment 3 {dm2}

kabin3 = Papp*SAin3 ; absorption rate constant of intestine compartment 3 {L/hr}

kin3 = kin ; transfer rate to intestine compartment 4 {/hr}

Vin4 = Vin ; volume of intestine compartment 4 {L}

SAin4 = SAin ; surface area of intestine compartment 4 {dm2}

kabin4 = Papp*SAin4 ; absorption rate constant of intestine compartment 4 {L/hr}

kin4 = kin ; transfer rate to intestine compartment 5 {/hr}

Vin5 = Vin ; volume of intestine compartment 5 {L}

SAin5 = SAin ; surface area of intestine compartment 5 {dm2}

kabin5 = Papp*SAin5 ; absorption rate constant of intestine compartment 5 {L/hr}

kin5 = kin ; transfer rate to intestine compartment 6 {/hr}

Vin6 = Vin ; volume of intestine compartment 6 {L}

SAin6 = SAin ; surface area of intestine compartment 6 {dm2}

kabin6 = Papp*SAin6 ; absorption rate constant of intestine compartment 6 {L/hr}

kin6 = kin ; transfer rate to intestine compartment 7 {hr}

Vin7 = Vin ; volume of intestine compartment 7 {L}

SAin7 = SAin ; surface area of intestine compartment 7 {dm2}

kabin7 = Papp*SAin7 ; absorption rate constant of intestine compartment 7 {L/hr}

kin7 = kin ; transfer rate to co {/hr}

kfe = 0.0198 ; transfer rate to feces {/hr}

;=====================================================================

;Partition Coefficients

;=====================================================================

PF = 144.094

;fat/blood partition coefficient calculated using QPPR of DeJongh et al. (1997)

PL =4.39074

;liver/blood partition coefficient calculated using QPPR of DeJongh et al. (1997)

PR = 4.39074

; rapidly perfused tissue/blood partition coefficient calculated using QPPR of DeJongh et al. (1997)

PS = 1.11843

;slowly perfused tissue/blood partition coefficient calculated using QPPR of DeJongh et al. (1997)

;=====================================================================

;Kinetic parameters

;=====================================================================

;Metabolism liver

;metabolism of 17ß-estradiol, scaled maximum rate of metabolism

CLint = S9P*VL*(CLintS9*60*1E-6) {L/hr} ;Hepatic clearance

CLintS9 = 65.52 {ul/min/mg protein} ;Hepatic clearance derived from S9 fraction;

;male: 175

;female: 65.52

S9P = 87*1000 {mg/kg} ;mg protein in kg liver

;reference: (Chiu and Ginsberg, 2011)

;=====================================================================

;Run settings

;=====================================================================

;Molecular weight

MW = 272.38 ; Molecular weight 17β-estradiol

;oral dose

ODOSEmg1 = 0.02 {mg/kg bw} ; ODOSEmg1 = given oral dose in mg/kg bw

ODOSEumol2 = ODOSEmg1*1E-3/MW*1E6 {umol/ kg bw}

;ODOSEumol2 = given oral dose recalculated to umol/kg bw

ODOSEumol=ODOSEumol2*BW; ; ODOSEumol = umol given oral

;--------------------------------------------------------------------------------------------------------------------

;IV dose

IVDOSEmg1 = 0 {mg/kg bw} ; IVDOSEmg1 = given IV dose in mg/kg bw

IVDOSEumol2 = IVDOSEmg1*1E-3/MW*1E6 {umol/ kg bw}

;IVDOSEumol2 = given oral dose recalculated to umol/kg bw

IVDOSEumol=IVDOSEumol2*BW ; IVDOSEumol = umol given IV

;time

Starttime = 0 ; in hr

Stoptime = 4 ; in hr

DTMIN = 1e-6 ; minimum integration time (DT)

DTMAX = 0.0015 ; maximum integration time (DT)

;=====================================================================

;Model calculations

;=====================================================================

;needle

;ANe = amount in needle

ANe' = -kd*ANe

Init ANe = IVDOSEumol

kd=1000000 ;kd, the trasport rate from needle to blood

;--------------------------------------------------------------------------------------------------------------------

;intestines, divided in 7 compartments

;Ain1 = Amount 17β-estradiol in intestine compartment 1 (umol)

Cin1 = Ain1/Vin1

Ain1' = - kin1*Ain1

Init Ain1 = ODOSEumol

;Ain2 = Amount 17β-estradiol in intestine compartment 2 (umol)

Cin2 = Ain2/Vin2

Ain2' = kin1*Ain1 - kin2*Ain2 - kabin2*Cin2

Init Ain2 = 0

;Ain3 = Amount 17β-estradiol in intestine compartment 3 (umol)

Cin3 = Ain3/Vin3

Ain3' = kin2*Ain2 - kin3*Ain3 - kabin3*Cin3

Init Ain3 = 0

;Ain4 = Amount 17β-estradiol in intestine compartment 4 (umol)

Cin4 = Ain4/Vin4

Ain4' = kin3*Ain3 - kin4*Ain4 - kabin4*Cin4

Init Ain4 = 0

;Ain5 = Amount 17β-estradiol in intestine compartment 5 (umol)

Cin5 = Ain5/Vin5

Ain5' = kin4*Ain4 - kin5*Ain5 - kabin5*Cin5

Init Ain5 = 0

;Ain6= Amount 17β-estradiol in intestine compartment 6 (umol)

Cin6 = Ain6/Vin6

Ain6' = kin5*Ain5 - kin6*Ain6 - kabin6*Cin6

Init Ain6 = 0

;Ain7= Amount 17β-estradiol in intestine compartment 7 (umol)

Cin7 = Ain7/Vin7

Ain7' = kin6*Ain6 - kin7*Ain7 - kabin7*Cin7

Init Ain7 = 0

;Aco = Amount 17β-estradiol in colon (umol)

Aco' = kin7*Ain7- kfe*Aco

Init Aco = 0

ACco' = kin7*Ain7

Init ACco = 0 ; cumulative amount reaching colon

;--------------------------------------------------------------------------------------------------------------------

;feces

;AFA = amount 17β-estradiol in feces (umol)

AFe' = kfe*Aco

Init AFe = 0

;--------------------------------------------------------------------------------------------------------------------

;liver compartment

;AL = Amount 17β-estradiol in liver tissue, umol

AL' = QL*(CB - CVL) + kabin2*Cin2 + kabin3*Cin3 + kabin4*Cin4 + kabin5*Cin5 + kabin6*Cin6 + kabin7*Cin7 - AMint'

Init AL = 0

CL = AL/VL

CVL = CL/PL

;AMint = amount 17β-estradiol metabolized

AMint' = CLint*CVL {umol/min}

init AMint = 0

;--------------------------------------------------------------------------------------------------------------------

;fat compartment

;AF = Amount 17β-estradiol in fat tissue (umol)

AF' = QF*(CB-CVF)

Init AF = 0

CF = AF/VF

CVF = CF/PF

;--------------------------------------------------------------------------------------------------------------------

;tissue compartment richly perfused tissue

;AR = Amount 17β-estradiol in rapidly perfused tissue (umol)

AR' = QR*(CB-CVR)

Init AR = 0

CR = AR/VR

CVR = CR/PR

;--------------------------------------------------------------------------------------------------------------------

;tissue compartment slowly perfused tissue

;AS = Amount 17β-estradiol in slowly perfused tissue (umol)

AS' = QS*(CB-CVS)

Init AS = 0

CS = AS/VS

CVS = CS/PS

;--------------------------------------------------------------------------------------------------------------------

; blood compartment

;AB = Amount 17β-estradiol in blood (umol)

AB' = (kd*ANe + QF*CVF + QL*CVL + QS*CVS + QR*CVR - QC*CB)

Init AB = 0

CB = AB/VB

AUC' = CB ;umol*min/L

Init AUC = 0

;=====================================================================

;Mass balance calculations

;=====================================================================

Total = ODOSEumol + IVDOSEumol

Calculated = Ain1 + Ain2 + Ain3 + Ain4 + Ain5 + Ain6 + Ain7 + Aco + AFe + AL + AMint + AF + AS + AR + AB + ANe

ERROR=((Total-Calculated)/Total+1E-30)*100

MASSBBAL=Total-Calculated + 1

2.2 PBK model for bisphenol A, built with in vitro and in silico data

Species: Rat

;=====================================================================

;Physiological parameters

;=====================================================================

;tissue volumes

BW = 0.250 {Kg} ; body weight rat (variable, dependent on study)

VFc = 0.07 ; fraction of fat tissue reference: (Brown et al., 1997).

VLc = 0.034 ; fraction of liver tissue reference: (Brown et al., 1997).

VBc = 0.074 ; fraction of blood reference: (Brown et al., 1997).

VRc = 0.09-VLc ; fraction of rapidly perfused tissue reference: (Brown et al., 1997).

VSc=0.746-VFc ; fraction of slowly perfused tissue reference: (Brown et al., 1997).

VF = VFc*BW {L or Kg} ; volume of fat tissue (calculated)

VL = VLc*BW {L or Kg} ; volume of liver tissue (calculated)

VB = VBc*BW {L or Kg} ; volume of blood (calculated)

VR = VRc*BW {L or Kg} ; volume of richly perfused tissue (calculated)

VS = VSc*BW {L or Kg} ; volume of slowly perfused tissue (calculated)

;--------------------------------------------------------------------------------------------------------------------

;blood flow rates

QC = 15*BW^0.74 {L/hr} ; cardiac output reference: (Brown et al., 1997).

QFc = 0.07 ; fraction of blood flow to fat reference: (Brown et al., 1997).

QLc = 0.25 ; fraction of blood flow to liver reference: (Brown et al., 1997).

QRc = 0.76 - QLc ; fraction of blood flow to rapidly perfused tissue reference: (Brown et al., 1997).

QSc = 0.24 – QFc ; fraction of blood flow to slowly perfused tissue reference: (Brown et al., 1997).

QF = QFc*QC {L/hr} ; blood flow to fat tissue (calculated)

QL = QLc*QC {L/hr} ; blood flow to liver tissue (calculated)

QR = QRc*QC {L/hr} ; blood flow to rapidly perfused tissue (calculated)

QS = QSc*QC {L/hr} ; blood flow to slowly perfused tissue (calculated)

;--------------------------------------------------------------------------------------------------------------------

; Intestinal lumen volumes, surfaces, absorption rates, transfer rates

;Papp, Caco-2 = 20 ; (x1E-6 cm/sec), in vitro, from Caco-2 reference: (Yoshkawa et al. 2002)

PappCaco2=-4.69897 ; Log Papp, Caco-2

;Log (Papp,in vivo) = 0.6836*Log(PappCaco-2)-0.5579 reference: (Sun et al. 2002)

Papp=10^(0.6836*PappCaco2-0.5579)*3600/10

;apparent intestinal permeability coefficient in vivo {dm/hr}

Vin = 0.0012 ; volume for each compartment of intestines {L}

SAin = 0.134 ; surface area {dm2}

kin = 4.17 ; transfer rate to next compartment within the intestines {/hr}

;kabin1 = Papp*SAin ; absorption rate constant {L/hr}

Vin1 = Vin ; volume of intestine compartment 1 {L}

SAin1 = SAin ; surface area of intestine compartment 1 {dm2}

kabin1 = Papp*SAin1 ; absorption rate constant of intestine compartment 1 {L/hr}

kin1 = kin ; transfer rate to intestine compartment 2 {/hr}

Vin2 = Vin ; volume of intestine compartment 2 {L}

SAin2 = SAin ; surface area of intestine compartment 2 {dm2}

kabin2 = Papp*SAin2 ; absorption rate constant of intestine compartment 2 {L/hr}

kin2 = kin ; transfer rate to intestine compartment 3 {/hr}

Vin3 = Vin ; volume of intestine compartment 3 {L}

SAin3= SAin ; surface area of intestine compartment 3 {dm2}

kabin3 = Papp*SAin3 ; absorption rate constant of intestine compartment 3 {L/hr}

kin3 = kin ; transfer rate to intestine compartment 4 {/hr}

Vin4 = Vin ; volume of intestine compartment 4 {L}

SAin4 = SAin ; surface area of intestine compartment 4 {dm2}

kabin4 = Papp*SAin4 ; absorption rate constant of intestine compartment 4 {L/hr}

kin4 = kin ; transfer rate to intestine compartment 5 {/hr}

Vin5 = Vin ; volume of intestine compartment 5 {L}

SAin5 = SAin ; surface area of intestine compartment 5 {dm2}

kabin5 = Papp*SAin5 ; absorption rate constant of intestine compartment 5 {L/hr}

kin5 = kin ; transfer rate to intestine compartment 6 {/hr}

Vin6 = Vin ; volume of intestine compartment 6 {L}

SAin6 = SAin ; surface area of intestine compartment 6 {dm2}

kabin6 = Papp*SAin6 ; absorption rate constant of intestine compartment 6 {L/hr}

kin6 = kin ; transfer rate to intestine compartment 7 {hr}

Vin7 = Vin ; volume of intestine compartment 7 {L}

SAin7 = SAin ; surface area of intestine compartment 7 {dm2}

kabin7 = Papp*SAin7 ; absorption rate constant of intestine compartment 7 {L/hr}

kin7 = kin ; transfer rate to co {/hr}

kfe = 0.0198 ; transfer rate to feces {/hr}

;=====================================================================

;Partition Coefficients

;=====================================================================

PF = 91.7956

;fat/blood partition coefficient calculated using QPPR of DeJongh et al. (1997)

PL = 2.74221

;liver/blood partition coefficient calculated using QPPR of DeJongh et al. (1997)

PR = 2.74221

; rapidly perfused tissue/blood partition coefficient calculated using QPPR of DeJongh et al. (1997)

PS = 0.861839

;slowly perfused tissue/blood partition coefficient calculated using QPPR of DeJongh et al. (1997)

;=====================================================================

;Kinetic parameters

;=====================================================================

;Metabolism liver

;metabolism of 17ß-estradiol, scaled maximum rate of metabolism

CLint = S9P*VL*(CLintS9*60*1E-6) {L/hr} ;Hepatic clearance

CLintS9 = 431.6 {ul/min/mg protein} ;Hepatic clearance derived from S9 fraction;

;male: 392

;female: 431.6

S9P = 87*1000 {mg/kg} ;mg protein in kg liver

;reference: (Chiu and Ginsberg, 2011)

;=====================================================================

;Run settings

;=====================================================================

;Molecular weight

MW = 228.29 ; Molecular weight bisphenol A

;oral dose

ODOSEmg1 = 10 {mg/kg bw} ; ODOSEmg1 = given oral dose in mg/kg bw

ODOSEumol2 = ODOSEmg1*1E-3/MW*1E6 {umol/ kg bw}

;ODOSEumol2 = given oral dose recalculated to umol/kg bw

ODOSEumol=ODOSEumol2*BW; ; ODOSEumol = umol given oral

;--------------------------------------------------------------------------------------------------------------------

;IV dose

IVDOSEmg1 = 0 {mg/kg bw} ; IVDOSEmg1 = given IV dose in mg/kg bw

IVDOSEumol2 = IVDOSEmg1*1E-3/MW*1E6 {umol/ kg bw}

;IVDOSEumol2 = given oral dose recalculated to umol/kg bw

IVDOSEumol=IVDOSEumol2*BW ; IVDOSEumol = umol given IV

;time

Starttime = 0 ; in hr

Stoptime = 8 ; in hr

DTMIN = 1e-6 ; minimum integration time (DT)

DTMAX = 0.0015 ; maximum integration time (DT)

;=====================================================================

;Model calculations

;=====================================================================

;needle

;ANe = amount in needle

ANe' = -kd*ANe

Init ANe = IVDOSEumol

kd=1000000 ;kd, the trasport rate from needle to blood

;--------------------------------------------------------------------------------------------------------------------

;intestines, divided in 7 compartments

;Ain1 = Amount bisphenol A in intestine compartment 1 (umol)

Cin1 = Ain1/Vin1

Ain1' = - kin1*Ain1

Init Ain1 = ODOSEumol

;Ain2 = Amount bisphenol A in intestine compartment 2 (umol)

Cin2 = Ain2/Vin2

Ain2' = kin1*Ain1 - kin2*Ain2 - kabin2*Cin2

Init Ain2 = 0

;Ain3 = Amount bisphenol A in intestine compartment 3 (umol)

Cin3 = Ain3/Vin3

Ain3' = kin2*Ain2 - kin3*Ain3 - kabin3*Cin3

Init Ain3 = 0

;Ain4 = Amount bisphenol A in intestine compartment 4 (umol)

Cin4 = Ain4/Vin4

Ain4' = kin3*Ain3 - kin4*Ain4 - kabin4*Cin4

Init Ain4 = 0

;Ain5 = Amount bisphenol A in intestine compartment 5 (umol)

Cin5 = Ain5/Vin5

Ain5' = kin4*Ain4 - kin5*Ain5 - kabin5*Cin5

Init Ain5 = 0

;Ain6= Amount bisphenol A in intestine compartment 6 (umol)

Cin6 = Ain6/Vin6

Ain6' = kin5*Ain5 - kin6*Ain6 - kabin6*Cin6

Init Ain6 = 0

;Ain7= Amount bisphenol A in intestine compartment 7 (umol)

Cin7 = Ain7/Vin7

Ain7' = kin6*Ain6 - kin7*Ain7 - kabin7*Cin7

Init Ain7 = 0

;Aco = Amount bisphenol A in colon (umol)

Aco' = kin7*Ain7- kfe*Aco

Init Aco = 0

ACco' = kin7*Ain7

Init ACco = 0 ; cumulative amount reaching colon

;--------------------------------------------------------------------------------------------------------------------

;feces

;AFA = amount bisphenol A in feces (umol)

AFe' = kfe*Aco

Init AFe = 0

;--------------------------------------------------------------------------------------------------------------------

;liver compartment

;AL = Amount bisphenol A in liver tissue, umol

AL' = QL*(CB - CVL) + kabin2*Cin2 + kabin3*Cin3 + kabin4*Cin4 + kabin5*Cin5 + kabin6*Cin6 + kabin7*Cin7 - AMint'

Init AL = 0

CL = AL/VL

CVL = CL/PL

;AMint = amount bisphenol A metabolized

AMint' = CLint*CVL {umol/min}

init AMint = 0

;--------------------------------------------------------------------------------------------------------------------

;fat compartment

;AF = Amount bisphenol A in fat tissue (umol)

AF' = QF*(CB-CVF)

Init AF = 0

CF = AF/VF

CVF = CF/PF

;--------------------------------------------------------------------------------------------------------------------

;tissue compartment richly perfused tissue

;AR = Amount bisphenol A in rapidly perfused tissue (umol)

AR' = QR*(CB-CVR)

Init AR = 0

CR = AR/VR

CVR = CR/PR

;--------------------------------------------------------------------------------------------------------------------

;tissue compartment slowly perfused tissue

;AS = Amount bisphenol A in slowly perfused tissue (umol)

AS' = QS*(CB-CVS)

Init AS = 0

CS = AS/VS

CVS = CS/PS

;--------------------------------------------------------------------------------------------------------------------

; blood compartment

;AB = Amount bisphenol A in blood (umol)

AB' = (kd*ANe + QF*CVF + QL*CVL + QS*CVS + QR*CVR - QC*CB)

Init AB = 0

CB = AB/VB

AUC' = CB ;umol*min/L

Init AUC = 0

;=====================================================================

;Mass balance calculations

;=====================================================================

Total = ODOSEumol + IVDOSEumol

Calculated = Ain1 + Ain2 + Ain3 + Ain4 + Ain5 + Ain6 + Ain7 + Aco + AFe + AL + AMint + AF + AS + AR + AB + ANe

ERROR=((Total-Calculated)/Total+1E-30)*100

MASSBBAL=Total-Calculated + 1
