# Supplementary material for: Towards a generic physiologically based kinetic model to predict in vivo uterotrophic responses in rats by reverse dosimetry of in vitro estrogenicity data
Source: Arch Toxicol. 2017 Dec 12;92(3):1075–88. doi: 10.1007/s00204-017-2140-5 (PMC5866837; doi:10.1007/s00204-017-2140-5)
Supplement: Supplementary file 3 — Supplementary material 3 (DOCX 375 KB) [file 204_2017_2140_MOESM3_ESM.docx]

Towards a generic physiologically based kinetic model to predict in vivo uterotrophic responses in rats by reverse dosimetry of in vitro estrogenicity data

Mengying Zhang^a*^, Bennard van Ravenzwaay^a,b^, Eric Fabian^b^, Ivonne M.C.M. Rietjens^a^, Jochem Louisse^a^

^a^ Division of Toxicology, Wageningen University, Stippeneng 4, 6708 WE Wageningen, the Netherlands

^b^ Experimental Toxicology and Ecology, BASF SE, Z 470, 67056 Ludwigshafen, Germany

^*^ Corresponding author: E-mail: [mengying.zhang@wur.nl](mailto:mengying.zhang@wur.nl); Tel: +31 317486396

**Supplementary material 3. Results of substrate depletion**

To determine the hepatic clearance (CL_int_) of E2 and BPA, substrate depletion was used. The CL_int_ of the parent compounds from phase I and phase II metabolism was determined in incubations with liver S9 fraction from both male and female Sprague Dawley rats, in the presence of the relevant co-factors NADPH, UDPGA, PAPS and acetyl CoA.

Two approaches were applied to investigate whether the in vitro CL_int_ values derived from incubations for individual reactions (with individual co-factors) is similar as the CL_int_ values derived from incubations with all the co-factors together in one mixture. In the first approach, the parent compound was incubating with individual co-factors and liver S9 fraction. In the second approach all the co-factors were added together to one mixture and incubated with the parent compound and liver S9 fraction. The results of depletion curves of all the experiments are shown in Figure 3.1 (E2) and Figure 3.2 (BPA).


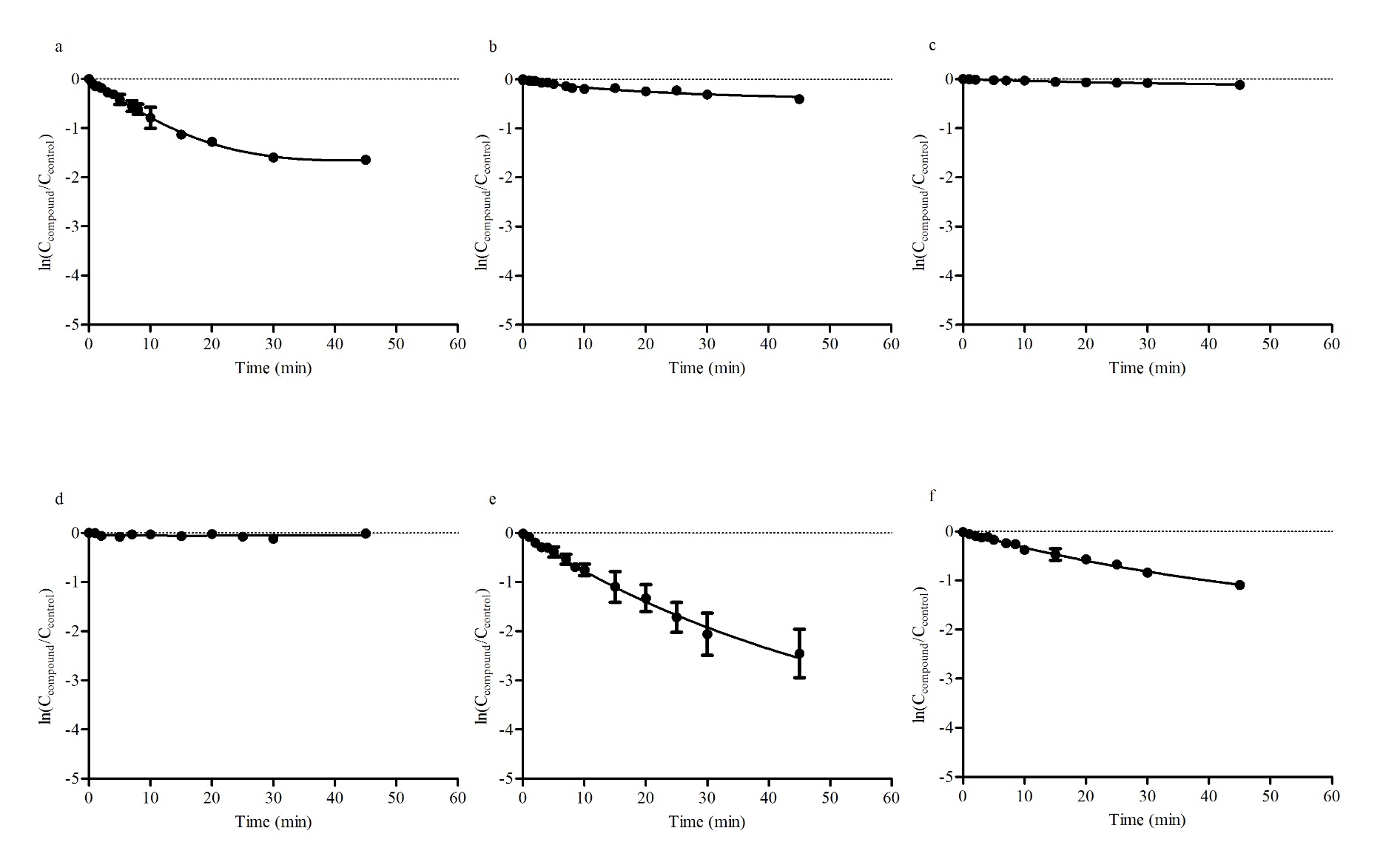


**Supplementary Fig. 3.1** The substrate depletion curves of E2. Symbols represent the average ln(C_compound_/C_control_) at different incubation time points. Lines represent the depletion curves of E2 derived from different incubation conditions. The parent compound E2 was incubated with: a) NADPH and liver S9 fraction from male Sprague-Dawley rats; b) UDPGA and liver S9 fraction from male Sprague-Dawley rats; c) PAPS and liver S9 fraction from male Sprague-Dawley rats; d) acetyl CoA and liver S9 fraction from male Sprague-Dawley rats; e) NADPH, UDPGA, PAPS and acetyl CoA as one mixture and liver S9 fraction from male Sprague-Dawley rats and f) NADPH, UDPGA, PAPS and acetyl CoA as one mixture and liver S9 fraction from female Sprague-Dawley rats.

**
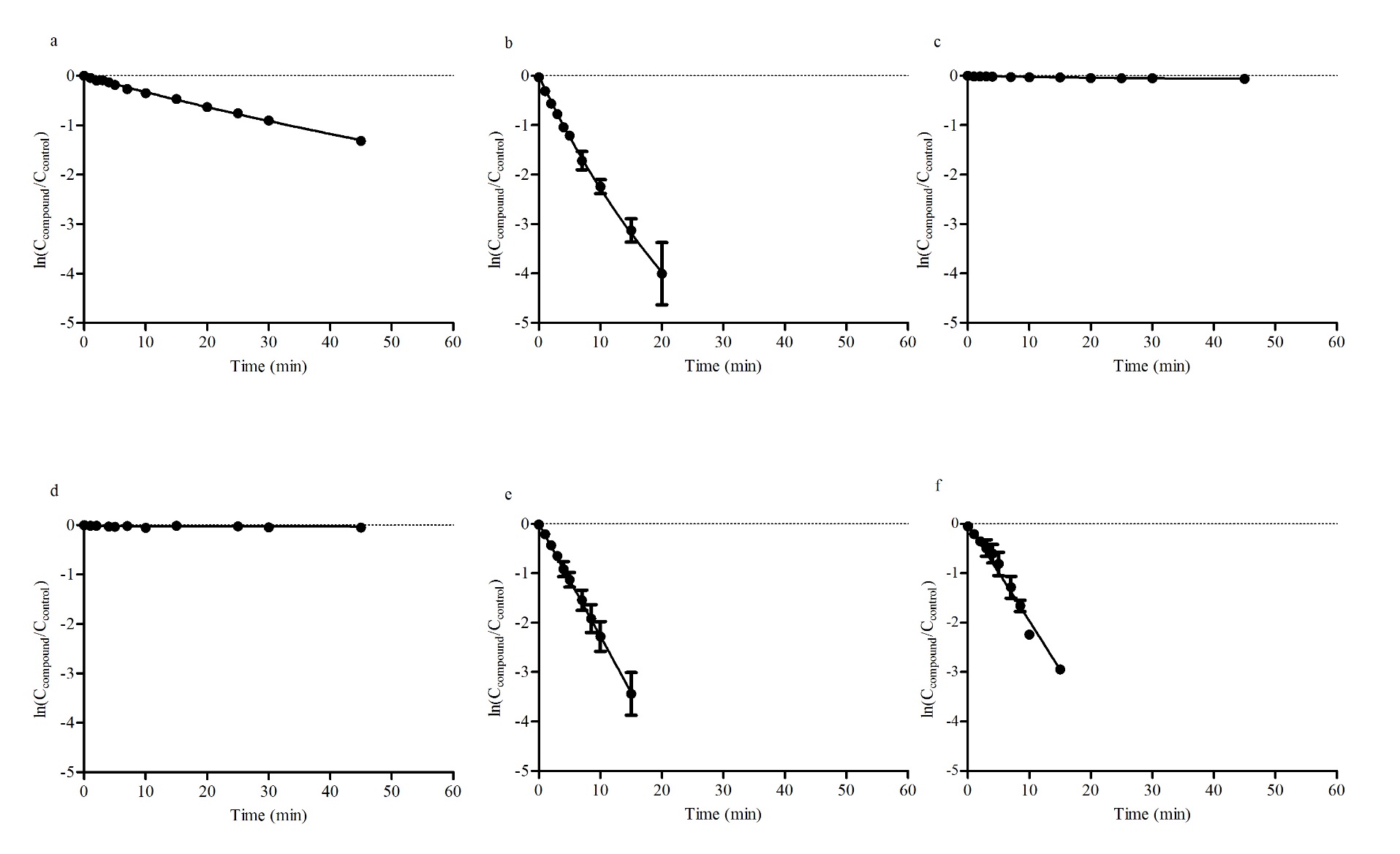
Supplementary Fig. 3.2** The substrate depletion curves of BPA. Symbols represent the average ln(C_compound_/C_control_) at different incubation time points. Lines represent the depletion curves of BPA derived from different incubation conditions. The parent compound BPA was incubated with: a) NADPH and liver S9 fraction from male Sprague-Dawley rats; b) UDPGA and liver S9 fraction from male Sprague-Dawley rats; c) PAPS and liver S9 fraction from male Sprague-Dawley rats; d) acetyl CoA and liver S9 fraction from male Sprague-Dawley rats; e) NADPH, UDPGA, PAPS and acetyl CoA as one mixture and liver S9 fraction from male Sprague-Dawley rats and f) NADPH, UDPGA, PAPS and acetyl CoA as one mixture and liver S9 fraction from female Sprague-Dawley rats.
