# Supplementary material for: Towards a generic physiologically based kinetic model to predict in vivo uterotrophic responses in rats by reverse dosimetry of in vitro estrogenicity data
Source: Arch Toxicol. 2017 Dec 12;92(3):1075–88. doi: 10.1007/s00204-017-2140-5 (PMC5866837; doi:10.1007/s00204-017-2140-5)
Supplement: Supplementary file 4 — Supplementary material 4 (DOCX 80 KB) [file 204_2017_2140_MOESM4_ESM.docx]

Towards a generic physiologically based kinetic model to predict in vivo uterotrophic responses in rats by reverse dosimetry of in vitro estrogenicity data

Mengying Zhang^a*^, Bennard van Ravenzwaay^a,b^, Eric Fabian^b^, Ivonne M.C.M. Rietjens^a^, Jochem Louisse^a^

^a^ Division of Toxicology, Wageningen University, Stippeneng 4, 6708 WE Wageningen, the Netherlands

^b^ Experimental Toxicology and Ecology, BASF SE, Z 470, 67056 Ludwigshafen, Germany

^*^ Corresponding author: E-mail: [mengying.zhang@wur.nl](mailto:mengying.zhang@wur.nl); Tel: +31 317486396

**Supplementary material 4. Results of BMD modelling**

BMD analysis was applied on the dose-response data using the exponential model for continuous data using the PROAST software from The National Institute for Public Health and the Environment of the Netherlands (RIVM) version 38.9 (Slob 2002).

When applying the data on the exponential model in the PROAST software, the benchmark response (BMR) corresponds to a 10% increase of response change compared to control (BMR_10_) in which the benchmark dose (BMD) is defined as the dose that gives a 10% extra risk (BMD_10_), the lower and upper limit of the 95% confidence interval on the benchmark dose at which a BMR equivalent to a 10% response change are shown as BMDL_10_ and BMDU_10_. The model provides BMD values only when data are adequately modelled.

We performed BMD modelling on two datasets of each chemical, the predicted dose-response data based on the YES assay data and the in vivo dose-response data. For the in vivo dose-response data set, responses are expressed as absolute uterus weight value (response-weight; see 4.1 for E2 and 4.3 for BPA). For the predicted dose-response data, responses were expressed as the percentage of the maximum response of the chemical (see 4.2 for E2 and 4.4 for BPA).

**4.1 BMD modelling on in vivo dose-response data of 17β-estradiol (E2)**

The in vivo uterus weight response data used for BMD modelling (Odum et al. 1997) is presented in Supplementary table 4.1.1. The results from PROAST are displayed in Supplementary table 4.1.3. All the results include the graphical representations of the fitted models.

**Supplementary table 4.1.1** In vivo data used for BMD modelling of uterus weight increase upon E2 exposure in rats (Odum et al., 1997).

| Dose (mg/kg bw) | Number of animals | Mean value of uterus weight (mg) | Standard deviation of uterus weight (mg) |
| --- | --- | --- | --- |
| 0 | 5 | 33.7 | 8.1 |
| 0.01 | 5 | 37.5 | 7.5 |
| 0.02 | 5 | 39 | 7.4 |
| 0.04 | 5 | 38.3 | 4.3 |
| 0.10 | 5 | 68.1 | 17.5 |
| 0.20 | 5 | 70.9 | 14.9 |
| 0.40 | 25 | 101.7 | 16.7 |

**Supplementary table 4.1.2** Results from the BMD analysis using PROAST software of the in vivo uterotrophic response data on absolute uterus weight in female AP rats exposed to 17β-estradiol (Odum et al. 1997). The table presents the benchmark dose (BMD_10_) for a BMR of a 10% uterus weight change compared to the control group with characteristics of the model fit.

| **Model type** | **BMR type** | **Log likelihood** | **Model accepted?** | **BMD** | **BMD** | |
| --- | --- | --- | --- | --- | --- | --- |
|  |  |  |  |  | **BMDL** | **BMDU** |
| Exponential | 10% | 14.96 | Yes | 0.0134 | 0.002915 | 0.033710 |


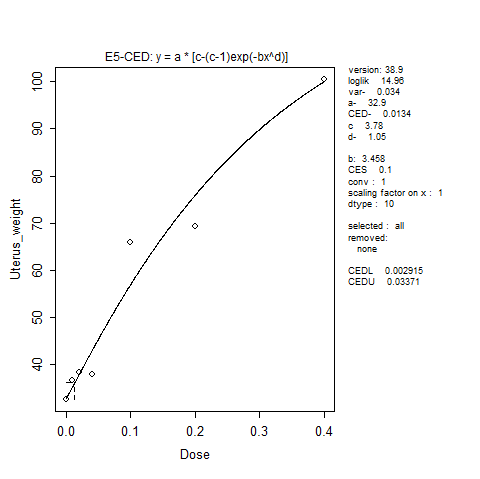


**4.2 BMD modelling on predicted dose-response data of E2**

BMD modelling was applied on the predicted dose-response data with the response presented as the percentage of the maximum response of the chemical. The predicted dose-response data used for BMD modelling are presented in Supplementary table 4.2.1. The results obtained from the PROAST software are presented in Supplementary table 4.2.2.

**Supplementary table 4.2.1** Predicted dose-response data of E2 used for BMD modelling. The response is shown as fold-induction compared to the control.

| Dose (mg/kg bw) | Numbers | Mean of response | Standard deviation |
| --- | --- | --- | --- |
| 0 | 3 | 2.0503 | 0.2128 |
| 7.32E-05 | 3 | 2.5056 | 0.1513 |
| 0.000732 | 3 | 2.5782 | 0.2674 |
| 0.007325 | 3 | 6.2337 | 1.5271 |
| 0.073246 | 3 | 68.4988 | 10.3407 |
| 0.732459 | 3 | 94.7687 | 5.1514 |
| 7.324591* | 3 | 94.7004 | 2.2109 |
| 73.24591* | 3 | 100 | 0 |

* The highest two dose groups were not applied in the BMD modelling.

**Supplementary table 4.2.2** Results from the BMD analysis using PROAST software of the predicted dose-response data of 17β-estradiol using PBK modelling-based reverse dosimetry of YES assay data. The table presents the benchmark dose (BMD_10_) for a BMR of a 10% response change compared to the control group with characteristics of the model fit.

| **Model type** | **BMR type** | **Log likelihood** | **Model accepted?** | **BMD** | **BMD** | |
| --- | --- | --- | --- | --- | --- | --- |
|  |  |  |  |  | **BMDL** | **BMDU** |
| Exponential | 10% | 11.63 | Yes | 0.00106 | 0.00074 | 0.00146 |


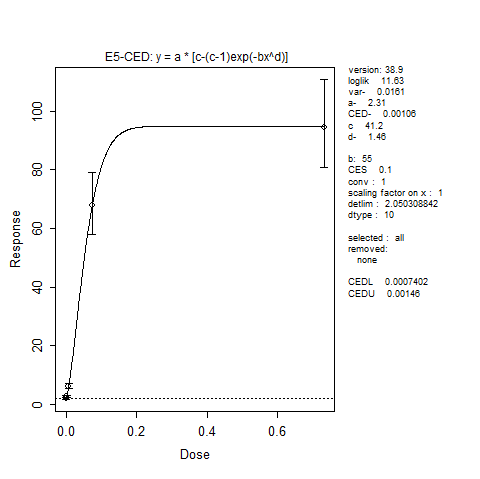


**4.3 BMD modelling on in vivo dose-response data of bisphenol A**

The in vivo data used for BMD modelling are presented in Supplementary table 4.3.1 (Kanno et al. 2003) and table 4.3.3 (Tinwell and Ashby 2004). The results from the BMD modelling using PROAST on the data of Kanno et al. (2003) are presented in Supplementary table 4.3.2 .The results from the BMD modelling using PROAST on the data of Tinwell and Ashby (2004) are presented in Supplementary table 4.3.4.

**Supplementary table 4.3.1** In vivo data used for BMD modelling of uterus weight increase upon BPA exposure in rats (Kanno et al. 2003).

| Dose (mg/kg bw) | Number of animals | Mean value of uterus weight (mg) | Standard deviation of uterus weight (mg) |
| --- | --- | --- | --- |
| 0 | 20 | 26.5 | 4.20 |
| 60 | 20 | 26.8 | 3.23 |
| 200 | 20 | 30.1 | 1.60 |
| 375 | 20 | 30.4 | 5.82 |
| 600 | 20 | 37.0 | 5.54 |
| 1000 | 20 | 44.1 | 8.36 |

**Supplementary table 4.3.2** Results from the BMD analysis using PROAST software of the in vivo uterotrophic response data on absolute uterus weight in female AP rats exposed to Bisphenol A (Kanno et al. 2003). The table presents the benchmark dose (BMD_10_) for a BMR of a 10% response change compared to the control group with characteristics of the model fit.

| **Model type** | **BMR type** | **Log likelihood** | **Model accepted?** | **BMD** | **BMD** | |
| --- | --- | --- | --- | --- | --- | --- |
|  |  |  |  |  | **BMDL** | **BMDU** |
| Exponential | 10% | 16.83 | Yes | 207 | 62.66 | 516.80 |


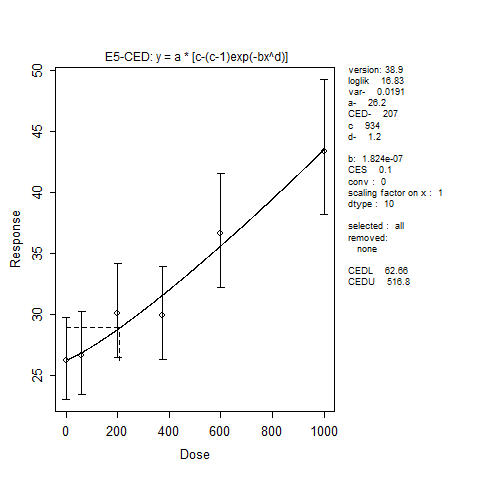


**Supplementary table 4.3.3** In vivo data used for BMD modelling of uterus weight increase upon BPA exposure in rats (Tinwell and Ashby 2004).

| Dose (mg/kg bw) | Number of animals | Mean value of uterus weight (mg) | Standard deviation of uterus weight (mg) |
| --- | --- | --- | --- |
| 0 | 5 | 19.4 | 2.1 |
| 10 | 5 | 23.9 | 3.3 |
| 100 | 5 | 29.0 | 3.8 |
| 300 | 5 | 38.5 | 6.3 |
| 600 | 5 | 41.4 | 7.8 |
| 800 | 5 | 59.0 | 8.6 |

**Supplementary table 4.3.4** Results from the BMD analysis using PROAST software of the in vivo uterotrophic response data on absolute uterus weight in female AP rats exposed to bisphenol A (Tinwell and Ashby 2004). The table presents the benchmark dose (BMD_10_) for a BMR of a 10% response change compared to the control group with characteristics of the model fit.

| **Model type** | **BMR type** | **Log likelihood** | **Model accepted?** | **BMD** | **BMD** | |
| --- | --- | --- | --- | --- | --- | --- |
|  |  |  |  |  | **BMDL** | **BMDU** |
| Exponential | 10% | 13.5 | Yes | 8.02 | 1.00 | 37.13 |


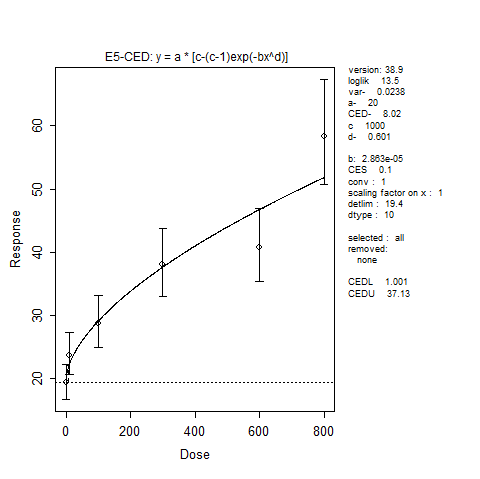


**4.4 BMD modelling on predicted dose-response data of BPA**

The BMD modelling was applied on the predicted dose-response data with the response presented as the percentage of the maximum response of the chemical. The data used for BMD modelling are presented in Supplementary table 4.4.1. The results obtained from PROAST software are displayed in Supplementary table 4.4.2.

**Supplementary table 4.4.1** Predicted dose-response data of BPA used for BMD modelling. The response is shown as fold-induction compared to the solvent control.

| Dose (mg/kg bw) | Numbers | Mean of response | Standard deviation |
| --- | --- | --- | --- |
| 0* | 3 | 5.9430 | 1.2060 |
| 0.002410097* | 3 | 6.0230 | 1.7480 |
| 0.024100968 | 3 | 5.8889 | 1.4935 |
| 0.241009676 | 3 | 6.2462 | 0.9294 |
| 2.410096763 | 3 | 6.4639 | 1.8691 |
| 24.10096763 | 3 | 7.9448 | 2.1794 |
| 241.0096763 | 3 | 39.2895 | 5.8835 |
| 2410.096763 | 3 | 100 | 0 |

*The first 2 dose groups were not included.

**Supplementary table 4.4.2** Results from the BMD analysis using PROAST software of the predicted dose-response data of bisphenol A using PBK modelling-based reverse dosimetry of YES assay data. The table presents the benchmark dose (BMD_10_) for a BMR of a 10% response change compared to the control group with characteristics of the model fit.

| **Model type** | **BMR type** | **Log likelihood** | **Model accepted?** | **BMD** | **BMD** | |
| --- | --- | --- | --- | --- | --- | --- |
|  |  |  |  |  | **BMDL** | **BMDU** |
| Exponential | 10% | 6.19 | Yes | 11.3 | 4.669 | 34.140 |


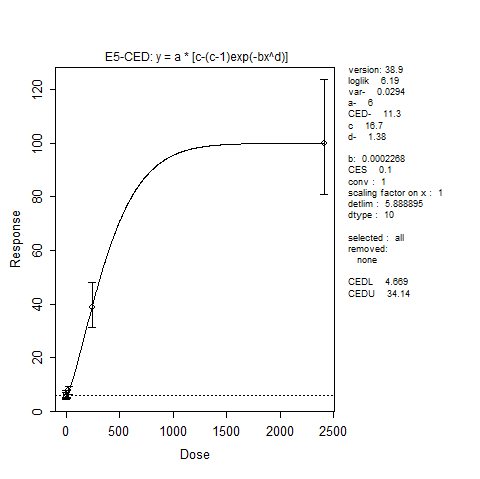


Kanno J, Onyon L, Peddada S, Ashby J, Jacob E, Owens W (2003) The OECD program to validate the rat uterotrophic bioassay. Phase 2: dose-response studies. Environmental health perspectives 111(12):1530

Odum J, Lefevre P, Tittensor S, et al. (1997) The rodent uterotrophic assay: critical protocol features, studies with nonyl phenols, and comparison with a yeast estrogenicity assay. Regulatory Toxicology and Pharmacology 25(2):176-188

Slob W (2002) PROAST: Software for dose-response modeling and benchmark dose analysis. RIVM.

Tinwell H, Ashby J (2004) Sensitivity of the immature rat uterotrophic assay to mixtures of estrogens. Environmental Health Perspectives 112(5):575
